# Fast autofluorescence imaging to evaluate dynamic changes in cell metabolism

**DOI:** 10.1117/1.JBO.29.12.126501

**Published:** 2024-12-19

**Authors:** Anna Theodossiou, Jocelyn Martinez, Alex J. Walsh

**Affiliations:** Texas A&M University, Department of Biomedical Engineering, College Station, Texas, United States

**Keywords:** nicotinamide adenine dinucleotide (phosphate), flavin adenine dinucleotide, live cell imaging, fluorescence microscopy, autofluorescence, cellular metabolism

## Abstract

**Significance:**

Cellular metabolic dynamics can occur within milliseconds, yet there are no optimal tools to spatially and temporally capture these events. Autofluorescence imaging can provide metabolic information on the cellular level due to the intrinsic fluorescence of reduced nicotinamide adenine dinucleotide (phosphate) [NAD(P)H] and flavin adenine dinucleotide (FAD).

**Aim:**

Our goal is to build and evaluate a widefield microscope optimized for rapid autofluorescence imaging of metabolic changes in cells.

**Approach:**

A widefield, fluorescence microscope was assembled from an inverted microscope base, an light-emitting diode (LED) for excitation, and an image splitter for simultaneous but separate imaging of two bandwidths of emission (451/106 and 560/94 nm) on a single scientific complementary metal–oxide–semiconductor (sCMOS) camera. MCF-7 cells and primary murine hippocampal neurons were metabolically perturbed using cyanide and imaged to optimize illumination and camera exposure. To capture a rapid change in metabolism, MCF-7 cells were starved for 1 h and imaged while reintroduced to glucose.

**Results:**

Significant differences in the optical redox ratio (ORR) and intensity of NAD(P)H divided by the summed intensities of NAD(P)H and FAD were quantified for cyanide-treated neurons and MCF-7 cells at illumination powers above 0.30 mW and camera exposures as low as 5 ms; however, low illumination and camera exposures hindered the ability to identify subcellular features. Minimal photobleaching was quantified for 30 s of continuous imaging for illuminations at 4.14 mW and below. Using the optimized illumination power of 4.14 mW and an exposure of 10 ms, continuous autofluorescence imaging of starved MCF-7 cells demonstrated a rapid, yet heterogeneous, increase in the ORR of cells upon exposure to glucose.

**Conclusions:**

Ultimately, this widefield autofluorescence imaging system allowed for dynamic imaging and quantification of cellular metabolism at 99.6 Hz.

## 1 Introduction

Metabolism is linked with cellular function through the generation of adenosine triphosphate, which is an energy carrier molecule required for cellular activities, including ion transportation, intracellular signaling, cell motility, and cell proliferation.^1^^,^^2^ Furthermore, metabolism is a dynamic process that adapts to cell needs, the extracellular environment, and intracellular events.^3^^–^^9^ As there is a strong relationship between cellular metabolism and function, metabolic dysfunction is a characteristic of many diseases including cancer, neurodegeneration, and musculoskeletal diseases.^10^^–^^13^ Therefore, studies of cellular metabolism provide insight into disease development, severity, progression, and therapeutic response. Although metabolism is known to be a dynamic process, few technologies with cellular resolution are capable of capturing rapid metabolic changes that accompany rapid cell signaling, membrane polarization, drug responses, or immediate environmental changes.^14^^–^^18^ Measurements of metabolic dynamics may provide useful diagnostic or prognostic information. The goal of this research is to design and evaluate a widefield fluorescence microscope to image metabolic fluctuations within live cells within subsecond frame rates.

Traditional assessments for cellular metabolism include a wide array of chemical and protein analyses, such as electrophoresis, chromatography, and mass spectrometry.^19^ However, these techniques are unable to resolve metabolic changes at a single-cell level. Plate reader–based metabolic assays, including the Seahorse XF-96 instrument, provide a time-course analysis of cellular metabolism through the measurement of oxygen consumption rates and the extracellular acidification rate.^20^^,^^21^ Seahorse metabolic assay measurements require a bulk population (5000 to 250,000 cells/well) to obtain viable information.^20^^,^^22^ As such, these traditional metabolic assessment techniques are temporally restricted and lack the resolution to detect single cells, thus limiting the ability to resolve metabolic differences within cell populations on a millisecond timeline. Therefore, a live cell technique for the detection of metabolic perturbations within millisecond durations is needed to detect fast cellular processes that underlie disease progression and drug responses.

Autofluorescence imaging of the metabolic co-enzymes, reduced nicotinamide dinucleotide (NADH) and flavin adenine dinucleotide (FAD), provides a label-free technique to capture metabolic information from cells. NADH is present within the mitochondria and cytosol, where it is used in oxidative phosphorylation and glycolysis. FAD is localized within the mitochondria and is used during oxidative phosphorylation and the electron transport chain. NADH and FAD naturally fluoresce at peak excitation wavelengths around 330 to 360 nm and 360 to 465 nm, respectively.^23^^,^^24^ NADH emits fluorescence between 410 and 490 nm, and FAD emits fluorescence between 510 and 650 nm.^25^ In addition, NADPH, the phosphorylated form of NADH, has excitation and emission spectra that directly overlap with NADH. Because of this overlap, NADH and NADPH signals are indistinguishable from one another in autofluorescence imaging,^26^ and nicotinamide adenine dinucleotide (phosphate) [NAD(P)H] is used for the measured, combined signal of NADH and NADPH. Similarly, there can be contributions from other fluorophores such as flavin mononucleotide (FMN), lipamide dehydrogenase (LipDH), and NAD(P)H that overlap with FAD fluorescence.^27^^–^^29^ As NAD(P)H is the reduced form of the molecule and FAD is the oxidized form, the optical redox ratio (ORR), which is a ratiometric measurement of the NAD(P)H and FAD fluorescence intensities, provides an optical measurement of the chemical redox balance of a cell.^30^ Autofluorescence imaging of NAD(P)H and FAD has been used to identify metabolic differences in heterogeneous cell populations, stem cell differentiation, and responses to pharmaceuticals.^31^^–^^37^

Autofluorescence imaging of NAD(P)H and FAD is advantageous for assessing cellular metabolism because it is label-free, has subcellular resolution, is live-cell compatible, and maintains the spatial integrity of the sample. However, a majority of prior studies that use autofluorescence imaging to assess cellular metabolism do so at a single time point, measure NADH and FAD separately, or characterize slow phenomena, such as cancer drug responses, over minutes or days.^31^^,^^33^^–^^37^ Rapid fluctuations in NAD(P)H and FAD fluorescence intensities have been observed with neural activity for point measurements acquired at 200 Hz,^7^ motivating the need for advances in autofluorescence imaging. Two- to 4-Hz imaging of autofluorescence has shown metabolic responses to rapid temperature changes and heart pacing in hypoxic conditions.^9^^,^^38^^,^^39^ Yet, acquisition rates of 2 to 4 Hz may still not capture many physiological events, such as neural activity or intracellular signaling,^7^^,^^9^^,^^38^^–^^40^ which could be reported via autofluorescence imaging.

Acquiring autofluorescence images at subsecond frame rates requires a balance between image acquisition speed and retention of meaningful metabolic information. Here, a customized widefield fluorescence microscope is designed and evaluated to determine the minimum image integration time necessary to capture cellular metabolism information from the autofluorescence of cells. First, a widefield fluorescence microscope was designed with an image splitter and re-aligner for simultaneous, but spatially separate, imaging of two emission bandwidths (451/106 and 560/94 nm) with a single camera. Control and cyanide-exposed MCF-7 breast cancer cells and primary murine hippocampal neurons were imaged to determine the minimum camera exposure time and optimal illumination power to resolve metabolic perturbations. Subsequently, photobleaching was quantified during 30 s of continuous imaging. Using the optimized illumination power and minimum exposure, the autofluorescence of metabolite-starved cells was imaged at 99.6 Hz continuously before, during, and after exposure to a bolus of glucose to demonstrate autofluorescence imaging of a rapid, cellular metabolic response.

## 2 Materials and Methods

### 2.1 Image Acquisition Setup

**Fig. 1:**
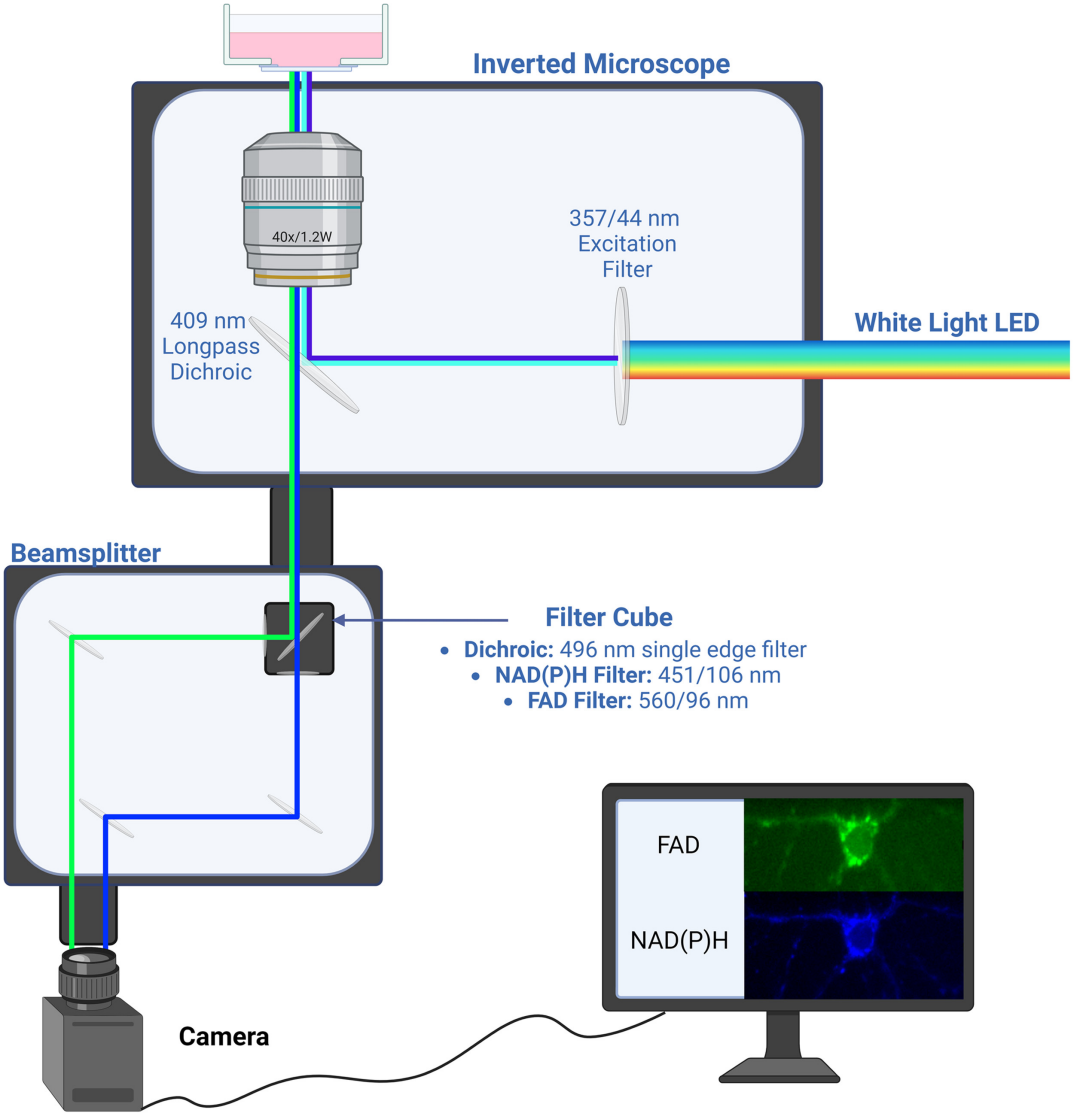
Illustration of imaging setup. White light LED is filtered and then used to excite autofluorescence; 451/106- and 560/94-nm emission wavelengths are then spectrally separated and recombined with a beam splitter to produce an image split by wavelength on the camera. Example images are 451- and 560-nm autofluorescence of primary murine hippocampal neurons. Image created with Ref. 41.

To achieve fast imaging, a fluorescence microscope was optimized for simultaneous imaging of 451- and 560-nm autofluorescence as shown in Fig. 1. An inverted microscope base (Zeiss, Axio-Observer Z1, Oberkochen, Germany) was used to simplify the illumination and detection light paths. For illumination, a white light-emitting diode (LED) (X-Cite Xylis, XT720S) was coupled to the back port of the microscope via a liquid light guide. A 357/44-nm filter (Brightline, FF01-357/44) was used in the microscope filter turret to filter the white-light LED to simultaneously excite NAD(P)H and FAD. To allow for spectral separation of blue and green emission wavelengths, a beam splitter (Cairn Research, OptoSplit II, Faversham, United Kingdom) was attached to the right port of the microscope base. A filter cube containing a 496-nm edge dichroic filter (SemRock^®^, FF496-SDi01-25x36x2.0) was placed within the beam splitter, allowing transmission of blue fluorescence while reflecting green fluorescence signal to a mirror. Additional filters, 451/106 nm (SemRock^®^, FF01-451/106-25) and 560/94 nm (SemRock^®^, FF01-560/94-25), were placed in the light path between the beam splitter and the camera to further refine the autofluorescence. Images of 2048×2048  pixels were captured with a scientific complementary metal–oxide–semiconductor (sCMOS) (Teledyne Photometrics, Prime BSI Express Scientific CMOS, Tucson, Arizona, United States) camera. As shown in Fig. 1, emissions centered at 451 and 560 nm from the same field of view (FOV) were split and displayed in a single image. All images were acquired with a 40×/1.2  NA water objective which resulted in a 333×333-μm field of view without the beam splitter or 333×167-μm field of view, with 451- and 560-nm images each filling half the FOV equally.

### 2.2 Cell Culturing Procedures

A breast cancer cell line, MCF-7 [American Type Culture Collection (ATCC), HTB-22™, Alexandria, Minnesota, United States] was acquired from the ATCC and cultured according to their standard procedures. MCF-7 cells were plated on 35-mm glass-bottom Petri dishes 48 h before imaging. These cells were plated at a density of 100,000 to 200,000 cells per dish. Immediately before imaging, cells were washed once to remove floating cells and imaged in phosphate-buffered saline (PBS). To generate cultures of primary neurons, E18 hippocampal murine tissues were dissociated into single neurons using papain (Transnet XY, C57EHP, Cordova, Tennessee, United States) and mechanical disruption. Approximately 10,000 to 25,000 cells were plated on 35-mm glass glass-bottom Petri dishes pre-coated with a mixture consisting of 15% of a poly-d-lysine (Millipore, A-003-E, Burlington, Massachusetts, United States) and 85% 8.5 pH borate buffer (Sigma-Aldrich, B0394, St. Louis, Missouri, United States). Fifty milliliters of Neurobasal-A media (Gibco, 10888022, Waltham, Massachusetts, United States) supplemented with 10% B-27™ Supplement (Gibco, 17504044), 1% d-glucose (Gibco, 17504044), and 1% 10,000 U/mL penicillin–streptomycin (Gibco, 15140122) was conditioned in a flask with a glial monolayer and used as the neuron growth media. After 7 days, neurons developed axons and dendrites and were used for imaging.

### 2.3 Illumination Power Assessment on Metabolic Signal Retention

NAD(P)H and FAD images of MCF-7 cells were acquired at nine different illumination powers, from 0.30 to 18.29 mW at a 10-ms camera exposure. For each illumination group, 10 to 15 FOVs were imaged for each condition, with eight dishes used per study. To compare against the control, the cells from different imaging dishes were metabolically perturbed using sodium cyanide (Sigma-Aldrich, 380970). For cyanide exposure, 100  μL of the 2-mL PBS solution was replaced with 100  μL of a 40× stock solution of sodium cyanide to make a final concentration of 4 mM. Five minutes after adding the cyanide, the cells were imaged with the same illumination powers as the control group. Illumination measurements were recorded at the focal point of the objective using a power meter (Thorlabs, PMD100, Newton, New Jersey, United States).

### 2.4 Photobleaching Assessment

To assess photobleaching within the cells over longitudinal image acquisitions, autofluorescence images of MCF-7 cells were continuously and simultaneously imaged for 30 s using 10-ms frame exposures and a 99.4-Hz frame rate. Each image stack consisted of 3000 frames. The image size was cropped to 297×891  pixels to achieve fast image acquisition.^42^

### 2.5 Exposure Effects on Metabolic Imaging

For both MCF-7 cells and neurons, in preparation for imaging, the normal growth media was replaced with 2 mL of PBS (Gibco, 20012-027) to reduce the background fluorescence from the phenol red in the media. Images were acquired at seven different camera exposure times ranging from 5 to 500 ms using an illumination of 4.14 mW. For each group, 12 FOVs were imaged with 8 to 14 dishes per study. To compare against the control for differences in metabolic signals through the ORR, the cells were metabolically perturbed using 4-mM sodium cyanide (Sigma-Aldrich, 380970).

### 2.6 Dynamic Metabolic Imaging

Approximately 48 h after plating, the MCF-7 cell media was replaced with a media absent of d-glucose, l-glutamine, and sodium pyruvate (Gibco, A14430-01) to metabolically starve the cells. Autofluorescence images were acquired after an hour of introducing the starved media to the cells.^27^ After 1 h and immediately before imaging, the media was replaced with PBS to reduce the background signal from riboflavin in the Dulbecco’s modified Eagle medium media. Both 451- and 560-nm images were acquired continuously at 99.6 Hz for a total of 3000 frames while a perfusion system introduced the PBS–glucose media (Gibco 20012-027, supplemented with 50-mM glucose) within 8 s of imaging to activate a rapid metabolic response. Images were cropped to an image size of 1436×456  pixels using MicroManager, an image acquisition software, to reduce acquisition time among frames.^42^

### 2.7 Data Analysis of Autofluorescence Images

Images were analyzed at the whole image level to evaluate autofluorescence changes for illumination power and exposure differences. In ImageJ, a manual threshold was set by visual inspection to remove low-intensity pixels due to noise from the background and nucleus pixels while maximizing the signal from the cytoplasm.^43^ The mean fluorescence intensity values for each emission channel (451/106 and 560/94 nm) were calculated for the remaining pixels above the threshold in each image in ImageJ. The ORR was then calculated at the image level using Eq. (1) where 451 nm represents the mean intensity of the 451-nm image, and 560 nm represents the mean intensity of the 560-nm image [Eq. (1)]^44^

$$\mathrm{ORR}=\frac{451\;\mathrm{nm}}{451\;\mathrm{nm}+560\;\mathrm{nm}}.$$ (1)

To evaluate the differences in ORR between control and cyanide-treated cells, for each illumination power and image acquisition time, groups were tested for normal distribution using a Shapiro–Wilk test (Supplementary Material). If groups followed a normal distribution, a standard t-test with an alpha significance value of 0.05 was used to evaluate the difference in the means. If groups did not follow a normal distribution, a Mann–Whitney U-test with an alpha significance value of 0.05 was applied to determine significance. For time-lapse studies, cells were selectively chosen based on the visibility of distinct features, such as the cytoplasm and nucleus. A region of interest (ROI), consisting of one cell, was manually segmented in the 451-nm channel image, after which the same trace was also applied to the 560-nm image. This was done due to the higher signal in the 451-nm channel, which allowed better visualization of the cells for tracing. The ROI was automatically applied to all frames within the image, and the mean intensity was derived for each frame in both the 451- and 560-nm channels.

To assess photobleaching and dynamic imaging trends, 12 cells were traced on the 451-nm images, and the mean intensity of each cell was quantified over the time stack of images. The same cell traces were applied to the respective 560-nm image, and the mean 560-nm autofluorescence of each cell was quantified over the time stack of images. The ORR was calculated for each cell at each frame using the ORR equation. ^30^ For photobleaching trends, the first 10 frames and last 10 frames of the image stack for each segmented cell were averaged and used to calculate the percent difference due to photobleaching for each cell [Eq. (2)]

$$\frac{I_{\mathrm{initial}10\mathrm{average}}-I_{\mathrm{last}10\mathrm{average}}}{I_{\mathrm{initial}10\mathrm{average}}}\times 100.$$ (2)

## 3 Results

### 3.1 Optimization of Illumination Power

**Fig. 2:**
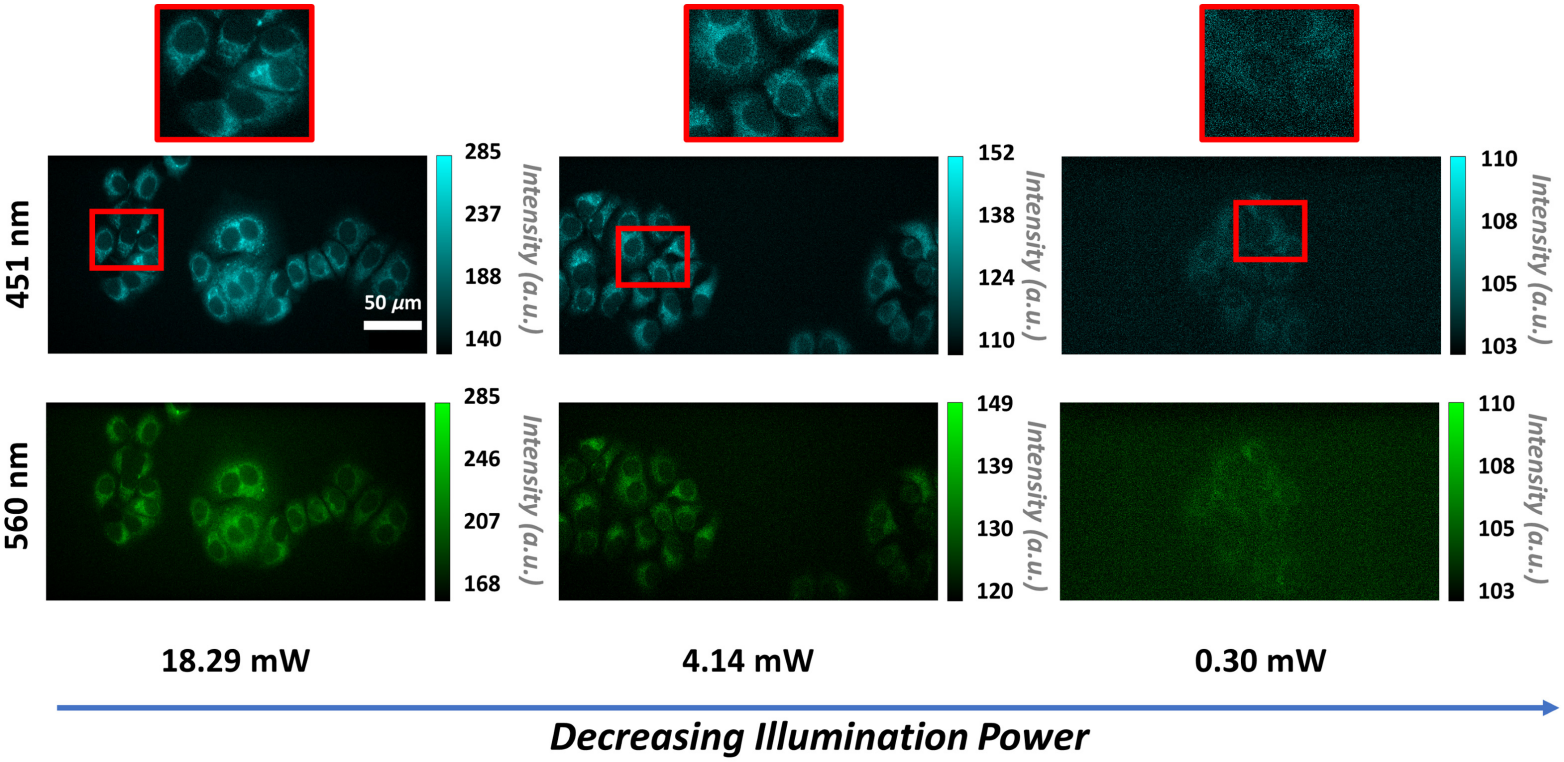
Representative 451- and 560-nm autofluorescence images of MCF-7 cells showing differences in signal for 18.29-, 4.14-, and 0.30-mW illumination powers at a 10-ms exposure time. Images are of MCF-7 cells without cyanide. As the illumination power is reduced, the intensity of 451- and 560-nm autofluorescence of the cells is also reduced. Scale bar is 50 *μ*m.

**Fig. 3:**
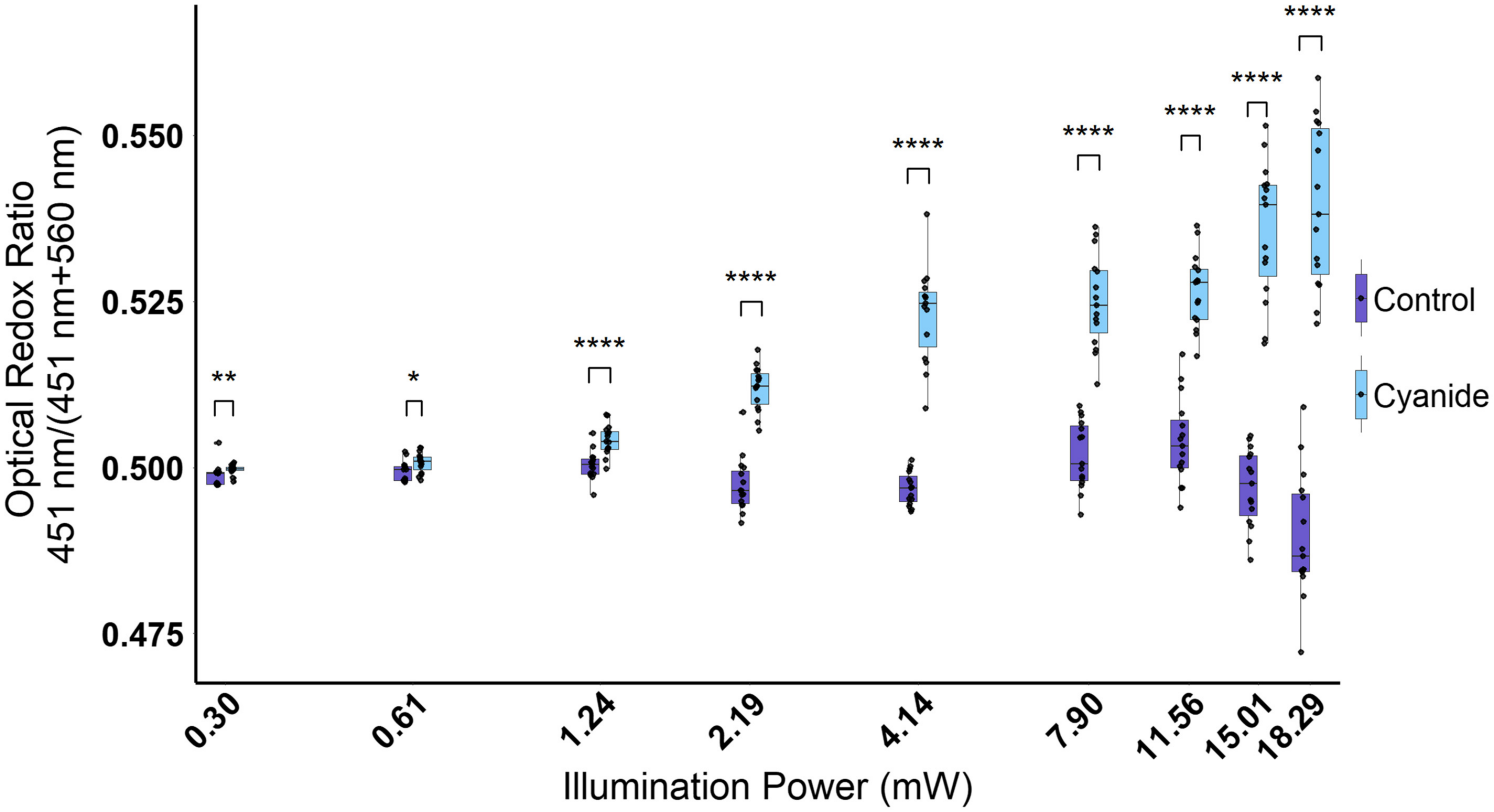
Boxplots of the optical redox ratio [451/(451 + 560)] nm of control and cyanide-treated MCF-7 cells at illumination powers 0.30 to 18.29 mW. The optical redox ratio is increased for the cyanide-treated cells compared with control cells at all illumination powers. *p<0.05, **p<0.01, ***p<0.001, and ****p<0.0001 for Student’s t-test or Mann–Whitney U-test of the means between the control and cyanide-treated groups. n=15 images per group, represented by each point on the boxplot.

First, a cyanide experiment was performed on MCF-7 cells using a consistent image acquisition time of 10 ms and variable illumination powers to determine the optimal excitation power for visualizing subcellular features and distinguishing metabolic states of control and cyanide-treated cells. Higher illumination powers allowed for better visualization of subcellular features, such as the cytoplasm and nucleus in both the 451- and 560-nm channels (Fig. 2). However, smaller structures, such as the mitochondria, were not identifiable at any of the illumination powers used throughout this assessment. At lower powers, specifically at 2.19 mW and below, features such as the cytoplasm and the nucleus became difficult to visualize (Fig. 2). As a result, the fluorescence of the cells was hard to distinguish from the background. Nevertheless, significant changes were quantified in the optical redox ratio between the control and cyanide-treated groups at all illumination powers (Fig. 3). When quantifying the ORR between the two different groups at each illumination power, the differences between the control and cyanide were greater at higher illumination powers. As the illumination decreased, the differences between the two groups were less noticeable (Fig. 3).

### 3.2 Assessment of Photobleaching

**Fig. 4:**
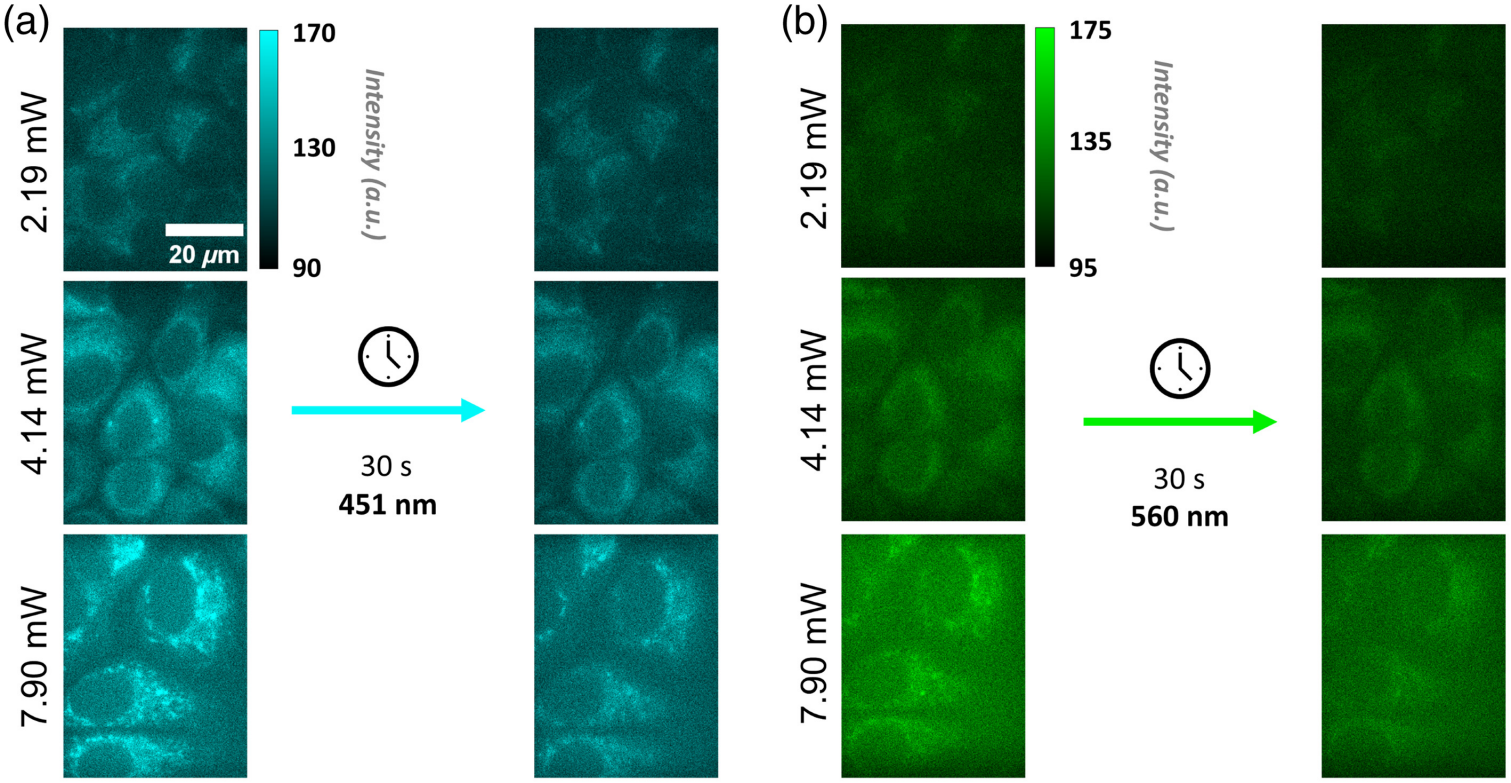
Representative 451-nm (a) and 560-nm (b) images of MCF-7 cells showing fluorescence signal at the beginning of the time series and after 30 s of continuous illumination with 2.19-, 4.14-, 7.90-, and 10-ms camera exposure time. Images are of MCF-7 cells without cyanide. At 4.14 mW, features, such as the cytoplasm and nucleus, are visualized at the end of the time series with minimal loss (5.57%) in signal due to photobleaching. Scale bar is 10  μm.

**Fig. 5:**
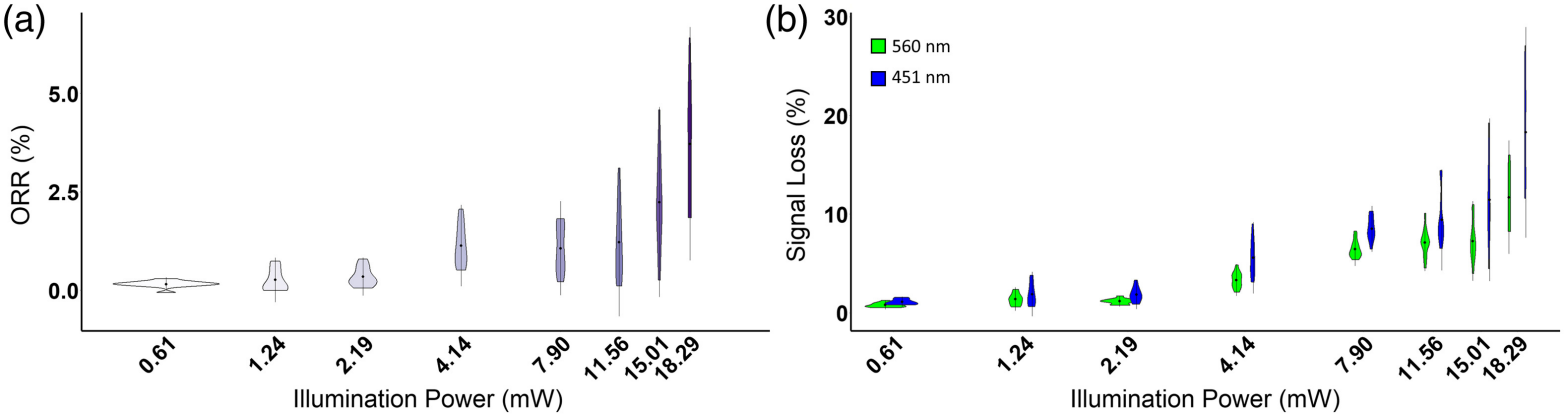
Violin plots depicting the percent change in (a) the ORR [451/(560 + 451)] nm and (b) 451 and 560 nm intensity values between the initial 10 frames and final 10 frames of 30 s of continuous imaging of MCF-7 cells. Images were acquired with illumination powers ranging from 0.61 to 18.29 mW. For each group, n=12 cells.

Although increased illumination power improves the contrast of the autofluorescence images, increased photobleaching due to higher illumination powers can compromise quantified metrics such as the optical redox ratio. Therefore, the change in fluorescence intensity due to photobleaching was assessed for illumination powers between 0.61 and 18.29 mW as MCF-7 cells were continuously imaged with constant excitation for 30 s. Over 30 s of imaging, the mean of both 451- and 560-nm fluorescence intensity values of the cells decreased, with increased changes in intensity for higher illumination powers (Fig. 4). Interestingly, the amount of photobleaching varied more across single cells at higher illumination powers (Fig. 5). However, cell morphology was hard to distinguish at lower powers (≤2.19  mW), even though these illumination powers exhibited the least amount of photobleaching. For illumination powers greater than 4.14 mW, some cells were not discernible in the FAD image at the end of the 30-s acquisition (Fig. 4). In the group illuminated with 4.14-mW excitation power, distinct cell morphology, such as the nucleus and the cytoplasm, was still identifiable at the end of the 30-s timeline. The average photobleaching changes within this group were minimal. In addition, this group in the 451-nm channel exhibited the highest signal-to-noise ratio (SNR) at the end time point (Table 1). At 4.14 mW, the ORR had less than a 1.13% change, 451 nm had less than 5.57% change, and 560 nm had less than 3.32% change over the 30-s timescale.

**Table 1:** Table of SNR values at each illumination power for the 451- and 560-nm channels.

SNR calculations						
	451-nm channel			560-nm channel		
Illumination power (mW)	Start SNR	End SNR	% change	Start SNR	End SNR	% change
0.61	53.8	54.0	0.4	51.7	52.5	1.5
1.24	53.1	52.7	−0.7	51.1	52.1	2.0
2.19	53.2	52.5	−1.4	51.0	51.6	1.1
4.14	56.5	55.2	−2.4	49.5	50.2	1.4
7.90	57.6	53.9	−6.4	51.1	49.3	−3.6
11.56	56.2	53.0	−5.7	50.1	49.2	−1.7
15.01	59.8	53.4	−10.7	51.0	48.2	−5.4
18.29	65.0	53.9	−17.1	55.7	49.9	−10.5

### 3.3 Minimum Exposure to Capture Metabolic Information in Autofluorescence Images

**Fig. 6:**
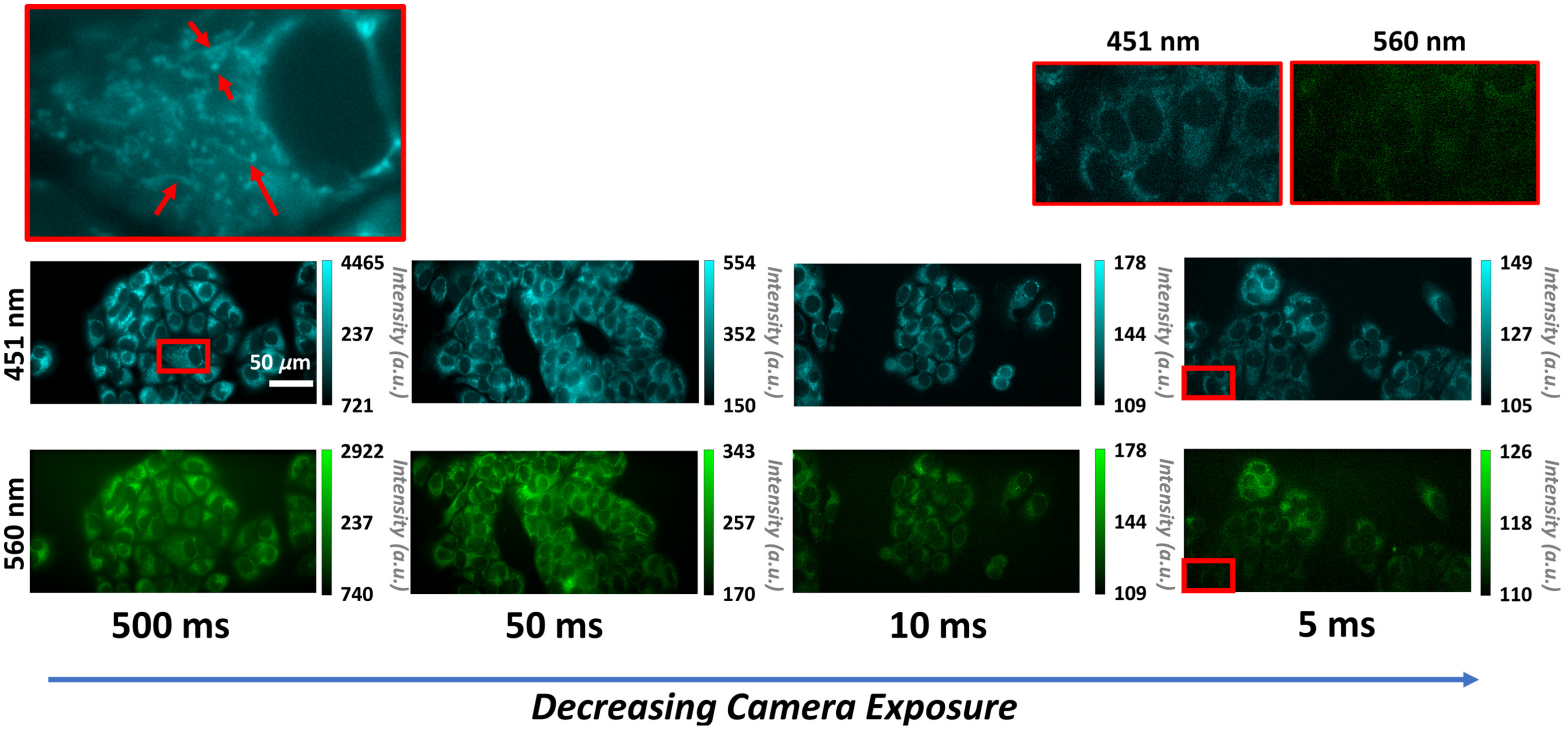
Representative 451- and 560-nm images of MCF-7 cells showing differences in intensity for 500-, 50-, 10-, and 5-ms camera acquisition durations at a 4.14-mW illumination power. Images are of MCF-7 cells without cyanide. The magnified image (left) highlights the ability to visualize mitochondrial structures. The magnified images (right) showcase the loss of visualization of subcellular structures in the 560-nm channel. As the camera acquisition time is reduced, the intensity of the autofluorescence of the cells is also reduced. Scale bar is 50  μm.

With the optimal illumination determined to be 4.14 mW, as this illumination was sufficient to visualize subcellular features, quantify metabolic changes, and minimize photobleaching, the camera exposure rate was studied next. Due to the localization of NADH in the mitochondria and cytosol, 451-nm autofluorescence images of cells demonstrate increased intensity throughout the cytosol region and a lower intensity within the nucleus (Fig. 6). Likewise, FAD is confined to the mitochondria, which can be illustrated by the localization of the bright pixels (Fig. 6). Although a lower camera exposure allows for faster image acquisition, less signal is detected by the camera sensor, resulting in reduced autofluorescence image intensities (Fig. 6). Within the images, however, cellular structures are identifiable at a 10-ms camera exposure time and above. A theoretical 0.16-μm pixel resolution allows visualization of subcellular features such as the nucleus and bright pixels corresponding to the mitochondria.^45^^,^^46^ However, at 5-ms image exposures for the control group, there is a loss of visualization of cells in the FAD channel (Fig. 6), even after applying a low-intensity threshold to remove background noise.

**Fig. 7:**
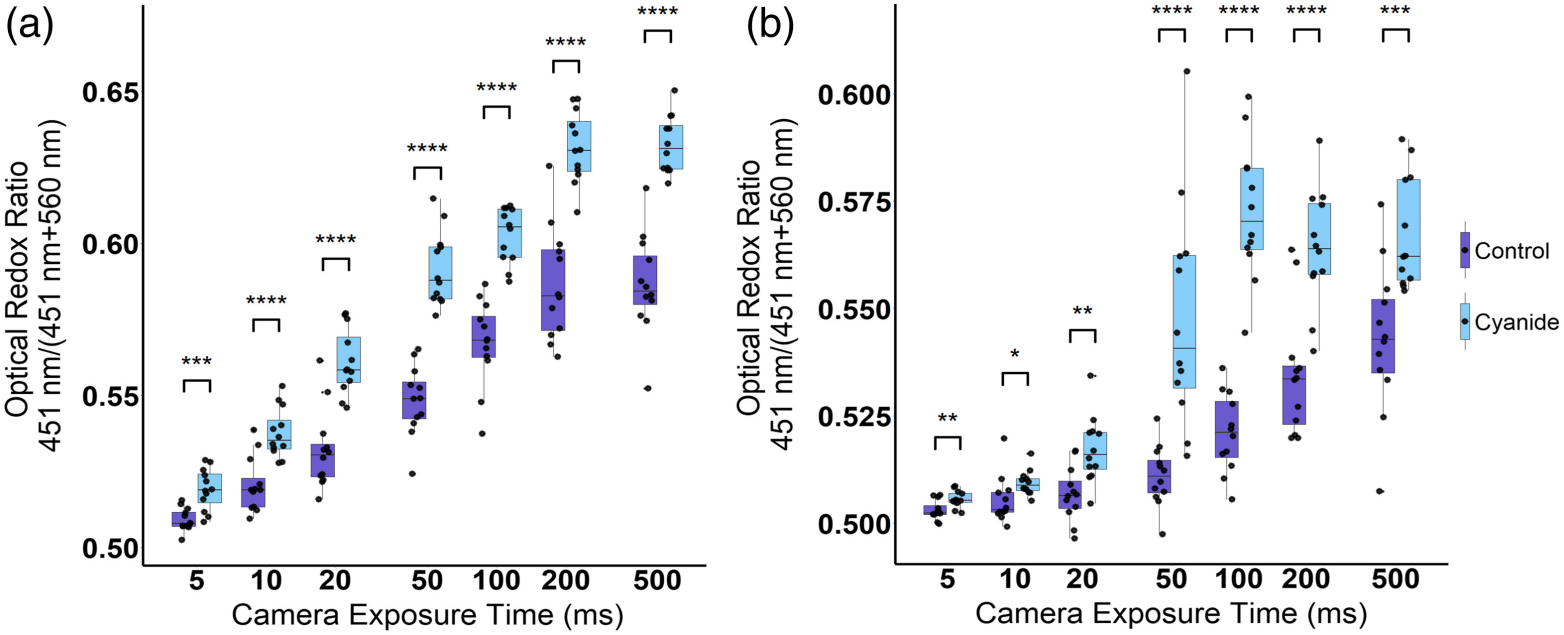
Boxplots of the optical redox ratio [451/(560 + 451)] nm of control and cyanide-treated MCF-7 (a) and E18 hippocampal murine neurons (b) for images acquired with camera acquisition times from 5 to 500 ms. The optical redox ratio is increased for the cyanide-treated cells compared with control cells within camera exposure times as low as 5 ms. *p<0.05, **p<0.01, ***p<0.001, and ****p<0.0001 for Student’s t-test or Mann–Whitney U-test of the means between the control and cyanide-treated groups. n=12 images per group, which is represented by each point.

A cyanide experiment was performed on MCF-7 and primary hippocampal neurons to determine the minimum camera exposure duration that retained metabolic differences between control and cyanide-treated cells. Across both cell lines, the ORR [451/(560 nm + 451 nm)] increases with increased camera acquisition duration (Fig. 7). These increases in the ORR could be attributed to different proportional effects of cyanide on the autofluorescence at the 451- and 560-nm channels. Another factor contributing to these differences could be the way manual thresholding was performed. At lower illumination powers and camera exposures, it becomes more difficult to isolate cell signals from the background and thus the background noise floor contributes a greater proportion to the overall mean intensity of the image. In addition, the ORR changes were statistically significant for exposures of 5 ms and above (Fig. 7).

### 3.4 Dynamic Metabolic Imaging

**Fig. 8:**
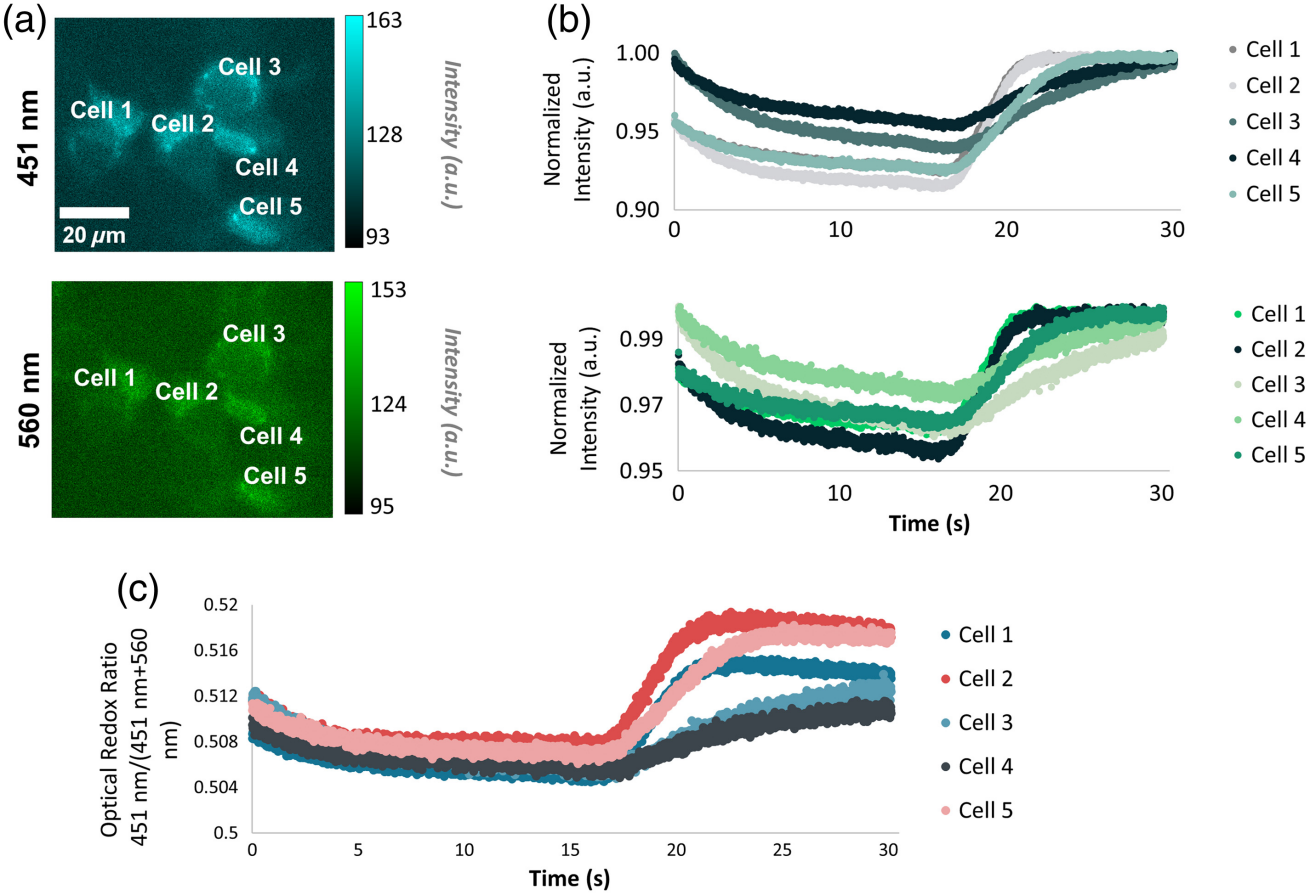
Representative 451- and 560-nm images of MCF-7 cells at a 4.14-mW illumination power (a). Changes in fluorescent signals are shown for 451 nm (b, top), 560 nm (b, bottom), and the ORR (c) for the five cells labeled. At around 8 s, glucose was introduced into the Petri dish to induce a heightened metabolic response. Images were acquired at a 10-ms camera exposure and a 99.6-Hz frame rate. Scale bar is 20  μm.

To validate that a 4.14-mW illumination power and 10 ms camera exposure time were suitable for imaging metabolic dynamics, MCF-7 cells were starved to induce a quiescent cell state. Then, glucose was introduced during imaging to elicit an increase in cellular metabolism. Before adding the PBS–glucose mixture, the autofluorescence intensity of the cells was low and decreased initially (451 nm: −3.1% to −5.8%; 560 nm: −0.8% to −3.5%), before the addition of glucose (Fig. 8). When the glucose-rich media was added to the Petri dish at ˜8s, there were delayed responses in both 451- and 560-nm fluorescent channels (8 to 9 s delay). In three out of the five cells, specifically cells 1, 2, and 5, 451-nm fluorescence increased rapidly (451 nm: 1.06 to 1.54 a.u./s; 560 nm: 0.43 to 1.12 a.u./s) following the addition of glucose and stayed elevated (Fig. 8) for the rest of the experiment. Interestingly, the 99.6-Hz imaging frame rate captured differences among the cells and their response to the glucose stimulus. Cells 1 and 2 reached their peak intensity before cell 5 (∼2.5 s faster). The 451-nm intensity of cells 3 and 4 also increased, yet at a slower rate (451 nm: 0.42 to 0.54 a.u./s; 560 nm: 0.22 to 0.27 a.u./s) than that of cells 1 and 2 (Fig. 8). Similar trends were observed for the 560-nm fluorescence channel. For cells 1, 2, and 5, the fluorescence intensity slightly increased once glucose was introduced to the media (∼2.0% change) (Fig. 8). The peak intensity was reached at slightly different time points for each of the cells, similar to the trends shown within the 451-nm channel (Fig. 8). These changes in fluorescence among the 451- and 560-nm channels caused different responses in the ORR among cells in the same FOV (Fig. 8).

## 4 Discussion and Conclusion

In this study, a widefield microscope was designed and optimized for rapid, simultaneous two-channel imaging of autofluorescence to capture dynamic metabolic events of cells. Because illumination power and camera exposure dictate the amount of signal detected, this study first explored the optimal imaging parameters for a customized wide-field fluorescence microscope to image and quantify meaningful metabolic information for dynamic imaging of glucose-starved MCF-7 cells. By assessing the ORR to detect cyanide-induced metabolic changes and qualitative features within the cells, an illumination power of 4.14 mW and a camera integration time of 10 ms were chosen. Using this imaging system at a 99.6-Hz frame rate, temporal and spatial differences in the autofluorescence and ORR of starved MCF-7 cells were captured due to a bolus glucose stimulus.

Signal detection and maximum frame rate to image autofluorescence will vary due to the specific optical components within a microscope system. Multiple camera sensors, including charge-coupled devices (CCD), electron-multiplying charge-coupled devices, and complementary metal-oxide-semiconductor (CMOS), can be used for fluorescence imaging applications.^47^^–^^49^ sCMOS cameras can achieve fast acquisition speeds while still maintaining a 95% quantum efficiency.^42^ A high quantum yield is ideal for imaging dynamic events with short image acquisitions and autofluorescence as the intrinsic fluorophores exhibit low emission signals. In comparison with traditional CMOS or CCD sensors, the sCMOS offers a larger field of view, lower noise levels, and faster frame rates with improved resolution.^47^^,^^49^ In addition, one camera paired with a beam splitter ensures perfect synchronization of the NADH and FAD images. Although multiple cells were able to be captured in the FOV, integrating a second camera would allow for a larger FOV to be imaged. It is likely that different cameras and light sources may alter the maximum illumination power and frame rate for imaging autofluorescence. Although one possible configuration that achieves 100-Hz frame rates is presented, the procedures to optimize imaging parameters provide a roadmap for determining the minimum frame rate of different systems.

The excitation and emission filters of the widefield microscope were selected for imaging NAD(P)H and FAD autofluorescence, based on reported absorption and emission spectra.^25^ Broad emission filters were chosen to maximize the fluorescence signal and minimize the required camera integration time. FAD in particular can yield low signals in cells due to its low quantum yield and low concentration.^28^^,^^50^ The 560/94-nm emission filter was chosen to detect as much FAD signal as possible; however, the broad emission bandwidth may enable the emission of additional fluorophores with similar spectral properties including FMN and LipDH.^29^ Furthermore, NAD(P)H has an emission tail that extends beyond 500 nm, so although the 451/106-nm filter was selected to maximize the NAD(P)H signal, some NAD(P)H signal may bleed through into the FAD channel during simultaneous imaging of the two fluorophores.^29^^,^^51^

When analyzing the fluorescence intensities of the two spectral channels individually, cyanide induced increased fluorescence in the 451-nm centered images as well as the 560-nm images of the MCF7 cells and for some groups of E18 primary murine hippocampal neurons. Although cyanide was expected to increase the fluorescence of the 451-nm channel as the primary fluorophore is NAD(P)H, it usually decreases FAD fluorescence, the expected contributor to the 560-nm channel.^27^^,^^52^^,^^53^ The inconsistent trends observed for the 560-nm images suggest the inclusion of additional fluorophores at this channel. Yet, the ORR trends for the cyanide (Figs. 3 and 7) and dynamic glucose experiments (Fig. 8) were consistent with previous experiments, demonstrating that this normalized metric can be used to detect changes in cellular metabolism from 451- to 560-nm autofluorescence.^27^^,^^52^^,^^53^ Here, in the results and discussion, rather than attribute each imaging channel to a specific fluorophore such as NAD(P)H and FAD, the channels are defined based on the center wavelength of the filter, 451 and 560 nm, respectively. Future studies could investigate emission filters with narrower bandwidths or a higher center wavelength for the green channel if improved isolation of NAD(P)H and FAD autofluorescence is desired.

One challenge with autofluorescence imaging is the weak quantum yields of the intrinsic fluorophores and their low abundance, both of which limit the fluorescence intensity. This issue can be mitigated through increased illumination or increased camera integration times. However, increased illumination power can induce photobleaching, which degrades the quality of the image and compromises quantified values, such as the ORR.^54^ Maximizing signal output while minimizing signal loss over the 30-s duration were important considerations to make when deciding on the optimal illumination power. At a 4.14-mW illumination power, the quality of the images was able to be preserved with minimal loss in overall signal, indicating low phototoxicity, and thus was chosen for this series of experiments (Fig. 4). During 30 s of continuous illumination, no morphological changes, such as cell blebbing, were observed for any of the cells of the time-series images. On the other hand, increased image acquisition duration prevents the capture of fast or dynamic changes associated with cellular metabolism, such as rapid cell signaling, membrane polarization, or immediate environmental changes.^14^^–^^18^

Cyanide perturbations are often used to validate optical microscopes for detecting metabolic information as cyanide disrupts the electron transport chain and leads to a noticeable increase in NADH.^55^^–^^58^ Therefore, cyanide perturbation was used here to determine the minimum exposures that still retain metabolic information. For statistical analysis, individual groups were treated as independent observations for each illumination power and frame rate as they were acquired from different imaging dishes, yet there may be some dependence within and across the groups as each sample was produced from the same cell culture. When optimizing the camera exposure, changes in cellular metabolism could be monitored at frame rates of at least 200 Hz in both MCF-7 cells and hippocampal neurons (Figs. 7 and 8).

Although quantitative assessment of the ORR shows significant changes induced by cyanide perturbations at all exposures, qualitative features are also important as visualization of individual cells and intracellular components such as mitochondria can provide additional information. As shown in Fig. 6, it was challenging to identify cells from the background noise within the 560-nm emission images acquired for 5 ms. Therefore, applying accurate cell traces in the FAD channel proved difficult. Imaging at the noise floor would be a problem for studies in which perturbations lower the 560-nm emission even further, as this would not be detected. Likewise, for studies in which quantification of individual cells is important, such as for assessing intra-group heterogeneity, the images must contain a high enough signal-to-noise ratio for segmentation of the cells. Because glucose starvation would initially decrease both 451- and 560-nm autofluorescent markers,^27^ a 10-ms camera integration time was suitable for the dynamic imaging studies. By assessing single-cell responses in MCF-7 cells, the study demonstrated differences in the rate of glucose uptake among neighboring cells Fig. 8. The varying responses of neighboring cells to glucose stimulation may correspond to basal differences in metabolism, mitochondrial membrane potential, cell cycle status, or GLUT1 expression.^59^^–^^62^ Previous assessments of metabolism have highlighted the importance of studying intracellular heterogeneity.^31^^,^^57^^,^^63^ This study demonstrates the ability to capture rapid, heterogeneous responses in MCF-7 and lays potential avenues for future studies of dynamic changes in cellular metabolism.

Dynamic changes in cellular metabolism were captured from autofluorescence imaging at 99.6 Hz, a significant improvement on previous studies, which were only able to achieve imaging with lower frame rates.^9^^,^^38^^,^^39^^,^^64^^–^^67^ Some of these studies used two-photon fluorescence lifetime imaging (2P-FLIM), which is intrinsically slower due to the laser-scanning nature of image collection, yet provides lifetime information, which can detect the amount of protein-bound and free versions of NAD(P)H and FAD.^28^ The long acquisition times are primarily attributed to time-correlated single-photon counting (TCSPC), which measures the fluorescence decay by detecting the arrival of single photons concerning the timing of the laser pulse.^68^ Widefield FLIM imaging either by a time-gated camera or in the frequency domain (FD) can achieve image speeds greater than single-point TCSPC methods; however, both methods have poor performance, low sensitivity, and limited lifetime resolution for low-signal fluorophores such as autofluorescence.^69^^,^^70^ Even though the widefield fluorescence microscopy system used herein is not able to measure the lifetime of NAD(P)H and FAD, widefield fluorescence imaging can achieve much faster acquisition times than 2P-FLIM while still preserving an FOV that captures multiple cells. Not only this, widefield imaging is able to achieve these high frames with a simple optical setup at a much lower cost relative to FLIM imaging.^53^ However, few studies have characterized the imaging parameters needed for fast imaging of NAD(P)H and FAD autofluorescence with widefield imaging.^28^ Even with the current studies utilizing widefield fluorescence microscopy to monitor fast events, acquisition times are much longer than what this study was able to achieve.^9^^,^^38^^,^^39^

Although cyanide induces a robust and repeatable metabolic perturbation for testing autofluorescence imaging systems to detect metabolic changes in cells, the stimulus is extreme. Even with an inability to see distinct structures, such as the cytoplasm and nucleus, in the control cells at low illumination powers and camera exposures (Figs. 2 and 4), the changes induced by cyanide were still able to be noted when quantifying the ORR in MCF-7 cells and hippocampal neurons (Figs. 3 and 7). Therefore, the optimal imaging parameters defined for a cyanide perturbation might not apply to all dynamic imaging studies using autofluorescence. Depending on the duration of the biological events of interest, different imaging timescales and illumination powers can be optimized for the specific application. Autofluorescence intensity changes can be correlated to relative changes in fluorophore concentrations, meaning the change in fluorescence signal will also vary depending on the metabolic event.^28^^,^^50^ Despite these experimental variables which can affect image quality, here, the process for optimizing the overall quality of images during dynamic autofluorescence studies is defined through systematic optimization of laser power, minimization of photobleaching, and determination of camera frame rate. Furthermore, the combination of autofluorescence imaging with post-processing methods such as deconvolution, machine learning, and denoising approaches could improve the overall SNR of time-lapse data and may enable even lower integration times and improve time-lapse frame rates.^71^^–^^73^

With the design and optimization of a widefield-fluorescent microscope, significant fluctuations in autofluorescence at 451 and 560 nm were able to be monitored simultaneously with a camera acquisition rate of 99.6 Hz and above. This system was able to monitor millisecond changes in the cellular metabolism of starved MCF-7 cells exposed to glucose. The ability to acquire images at 99.6 Hz allows the detection of changes in cellular metabolism that would otherwise be missed at slower acquisition rates.

## Supplementary Material



## Data Availability

All relevant data used in this research are available at https://github.com/walshlab/FastImaging
