# Supplementary material for: Fast autofluorescence imaging to evaluate dynamic changes in cell metabolism
Source: J Biomed Opt. 2024 Dec 19;29(12):126501. doi: 10.1117/1.JBO.29.12.126501 (PMC11657876; doi:10.1117/1.JBO.29.12.126501)

QQ-Plots for Illumination Power Assessment

**0.30 mW: No Cyanide**			
Shapiro Wilk Results: p=7.6992e-04, Not normally Distributed			
Rank	Percentile	Z Score	No cyanide
1	0.033333333	-1.833914636	0.497348389
2	0.1	-1.281551566	0.497384057
3	0.166666667	-0.967421566	0.497395759
4	0.233333333	-0.727913291	0.497412405
5	0.3	-0.524400513	0.497510121
6	0.366666667	-0.340694827	0.497612198
7	0.433333333	-0.167894005	0.49910967
8	0.5	0	0.499111578
9	0.566666667	0.167894005	0.499157184
10	0.633333333	0.340694827	0.499169602
11	0.7	0.524400513	0.499236415
12	0.766666667	0.727913291	0.499277267
13	0.833333333	0.967421566	0.499322674
14	0.9	1.281551566	0.499641174
15	0.966666667	1.833914636	0.503671507

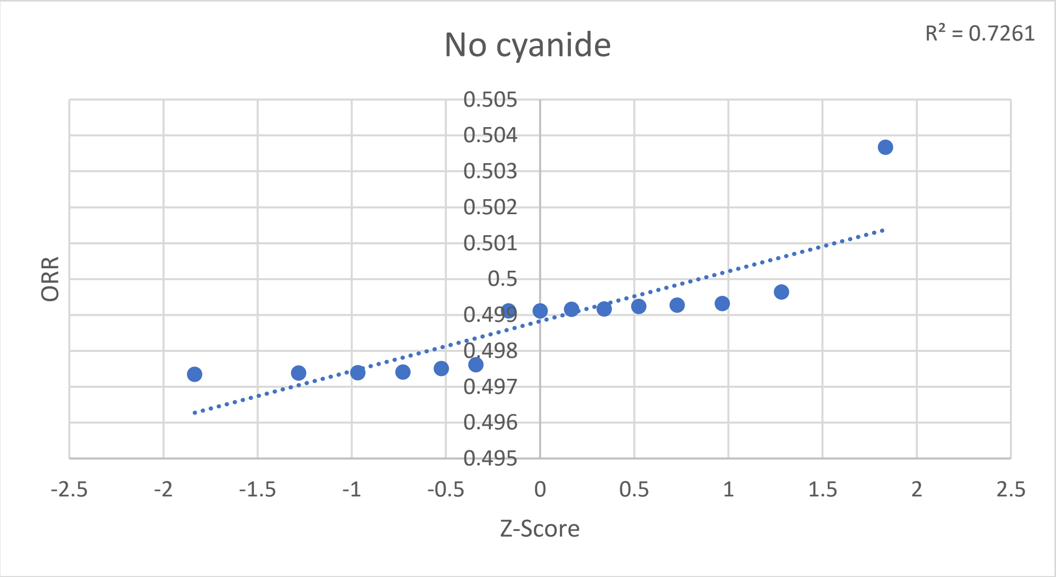

**0.30 mW: Cyanide**			
Shapiro Wilk Results: p=0.0315, Not normally Distributed			
Rank	Percentile	Z Score	Cyanide
1	0.033333333	-1.833914636	0.497807618
2	0.1	-1.281551566	0.498405482
3	0.166666667	-0.967421566	0.499395804
4	0.233333333	-0.727913291	0.499503134
5	0.3	-0.524400513	0.499573002
6	0.366666667	-0.340694827	0.499675331
7	0.433333333	-0.167894005	0.499727838
8	0.5	0	0.499735191
9	0.566666667	0.167894005	0.499790118
10	0.633333333	0.340694827	0.499881633
11	0.7	0.524400513	0.499928415
12	0.766666667	0.727913291	0.500142586
13	0.833333333	0.967421566	0.500311532
14	0.9	1.281551566	0.500539496
15	0.966666667	1.833914636	0.500700804

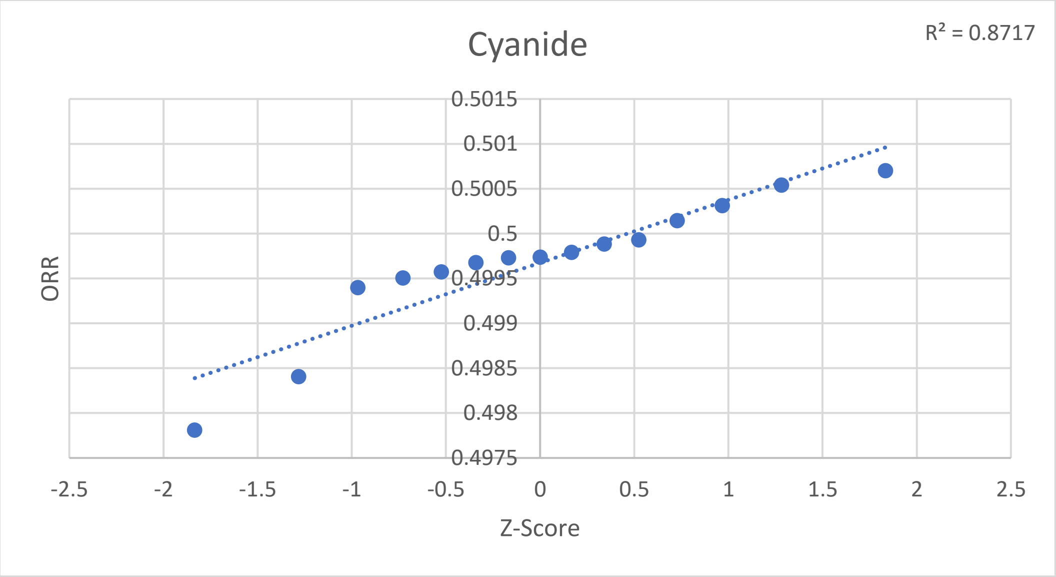

0.61 mW: No Cyanide

Shapiro Wilk Results: p=0.0316, Not normally Distributed

Rank	Percentile	Z Score	No cyanide
1	0.033333333	-1.833914636	0.497707437
2	0.1	-1.281551566	0.497899532
3	0.166666667	-0.967421566	0.497968683
4	0.233333333	-0.727913291	0.49798548
5	0.3	-0.524400513	0.498013954
6	0.366666667	-0.340694827	0.498095052
7	0.433333333	-0.167894005	0.498296498
8	0.5	0	0.499624994
9	0.566666667	0.167894005	0.499695442
10	0.633333333	0.340694827	0.500072476
11	0.7	0.524400513	0.500132118
12	0.766666667	0.727913291	0.5002154
13	0.833333333	0.967421566	0.500333417
14	0.9	1.281551566	0.501984265
15	0.966666667	1.833914636	0.502362589

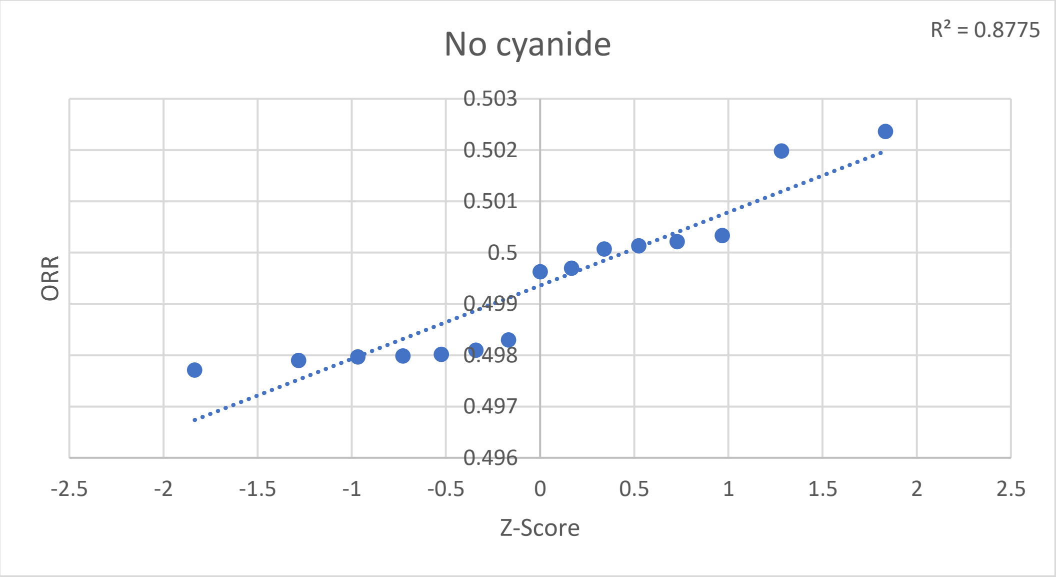

0.61 mW: Cyanide

Shapiro Wilk Results: p=0.4968, Normally Distributed

Rank	Percentile	Z Score	Cyanide
1	0.033333333	-1.833914636	0.49800678
2	0.1	-1.281551566	0.498503977
3	0.166666667	-0.967421566	0.498898852
4	0.233333333	-0.727913291	0.499094
5	0.3	-0.524400513	0.500189312
6	0.366666667	-0.340694827	0.50039704
7	0.433333333	-0.167894005	0.500823777
8	0.5	0	0.500933646
9	0.566666667	0.167894005	0.501109732
10	0.633333333	0.340694827	0.501401832
11	0.7	0.524400513	0.501456923
12	0.766666667	0.727913291	0.501687663
13	0.833333333	0.967421566	0.502495894
14	0.9	1.281551566	0.502893575
15	0.966666667	1.833914636	0.502898724

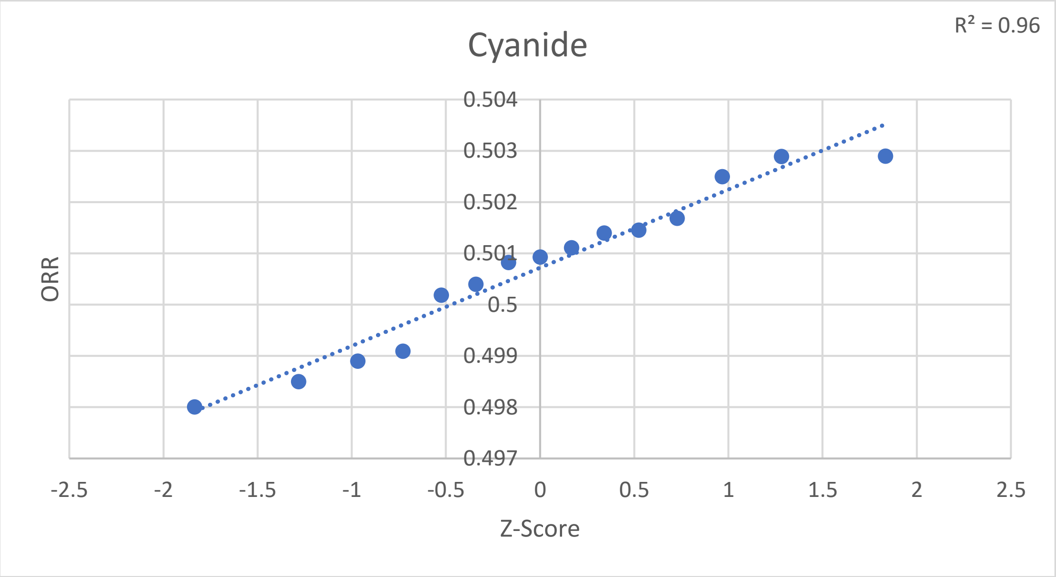

1.24 mW: No Cyanide

Shapiro Wilk Results: p=0.3160, Normally Distributed

Rank	Percentile	Z Score	No cyanide
1	0.033333333	-1.833914636	0.495873916
2	0.1	-1.281551566	0.498523345
3	0.166666667	-0.967421566	0.498762017
4	0.233333333	-0.727913291	0.498953543
5	0.3	-0.524400513	0.499095375
6	0.366666667	-0.340694827	0.499839981
7	0.433333333	-0.167894005	0.500047681
8	0.5	0	0.500424644
9	0.566666667	0.167894005	0.500760678
10	0.633333333	0.340694827	0.500807499
11	0.7	0.524400513	0.501111111
12	0.766666667	0.727913291	0.501468716
13	0.833333333	0.967421566	0.501469187
14	0.9	1.281551566	0.503126751
15	0.966666667	1.833914636	0.505148595

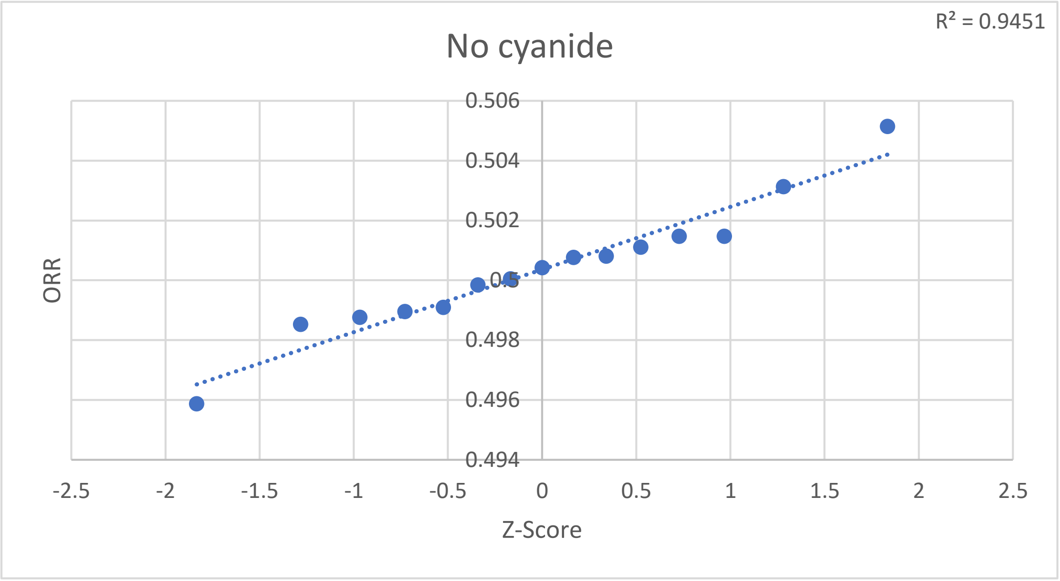

1.24 mW: Cyanide

Shapiro Wilk Results: p=0.9389, Normally Distributed

Rank	Percentile	Z Score	Cyanide
1	0.033333333	-1.833914636	0.499775008
2	0.1	-1.281551566	0.501086248
3	0.166666667	-0.967421566	0.502390621
4	0.233333333	-0.727913291	0.502704941
5	0.3	-0.524400513	0.502793196
6	0.366666667	-0.340694827	0.502944884
7	0.433333333	-0.167894005	0.50378094
8	0.5	0	0.503909686
9	0.566666667	0.167894005	0.504610917
10	0.633333333	0.340694827	0.505063817
11	0.7	0.524400513	0.505253917
12	0.766666667	0.727913291	0.505638675
13	0.833333333	0.967421566	0.50604603
14	0.9	1.281551566	0.507850656
15	0.966666667	1.833914636	0.507943731

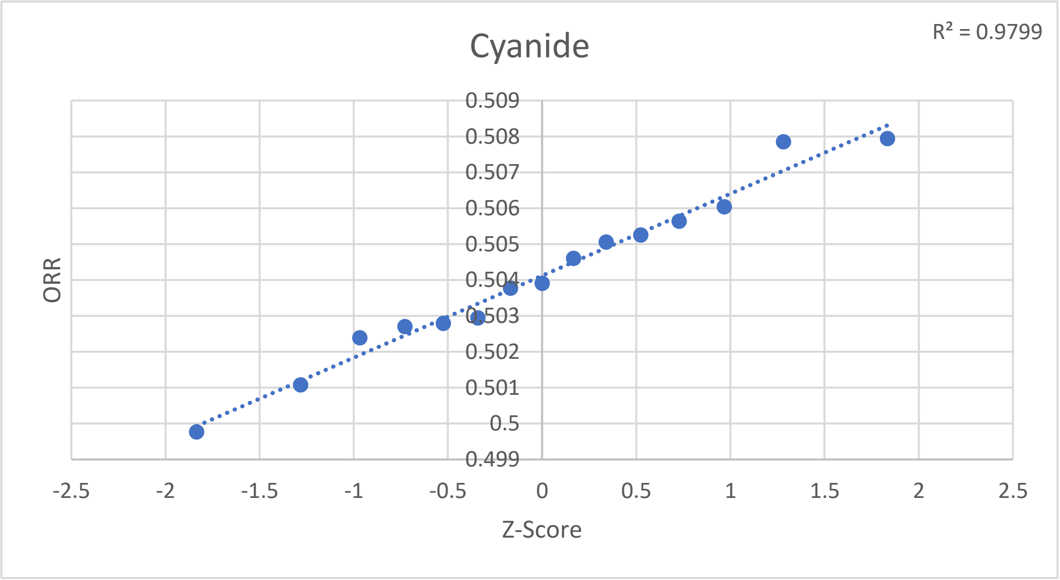

2.19 mW: No Cyanide			
Shapiro Wilk Results: p=0.0839, Normally Distributed			
Rank	Percentile	Z Score	No cyanide
1	0.033333333	-1.833914636	0.491577557
2	0.1	-1.281551566	0.492970269
3	0.166666667	-0.967421566	0.494304339
4	0.233333333	-0.727913291	0.494339574
5	0.3	-0.524400513	0.494832559
6	0.366666667	-0.340694827	0.49584735
7	0.433333333	-0.167894005	0.495890589
8	0.5	0	0.49648131
9	0.566666667	0.167894005	0.496487364
10	0.633333333	0.340694827	0.497786911
11	0.7	0.524400513	0.498976799
12	0.766666667	0.727913291	0.49991919
13	0.833333333	0.967421566	0.500204375
14	0.9	1.281551566	0.501815892
15	0.966666667	1.833914636	0.508311626

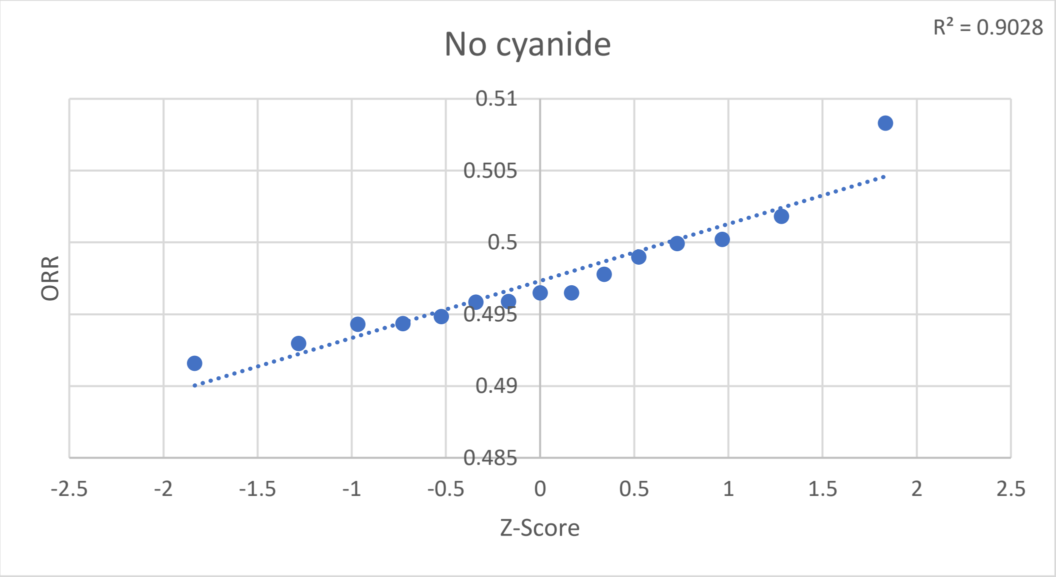

2.19 mW: Cyanide			
Shapiro Wilk Results: p=0.8504, Normally Distributed			
Rank	Percentile	Z Score	Cyanide
1	0.033333333	-1.833914636	0.505561257
2	0.1	-1.281551566	0.506790796
3	0.166666667	-0.967421566	0.508617659
4	0.233333333	-0.727913291	0.508952386
5	0.3	-0.524400513	0.510167822
6	0.366666667	-0.340694827	0.511985
7	0.433333333	-0.167894005	0.512098625
8	0.5	0	0.512225083
9	0.566666667	0.167894005	0.513109231
10	0.633333333	0.340694827	0.513363541
11	0.7	0.524400513	0.513620515
12	0.766666667	0.727913291	0.514600982
13	0.833333333	0.967421566	0.514632001
14	0.9	1.281551566	0.515555484
15	0.966666667	1.833914636	0.517735905

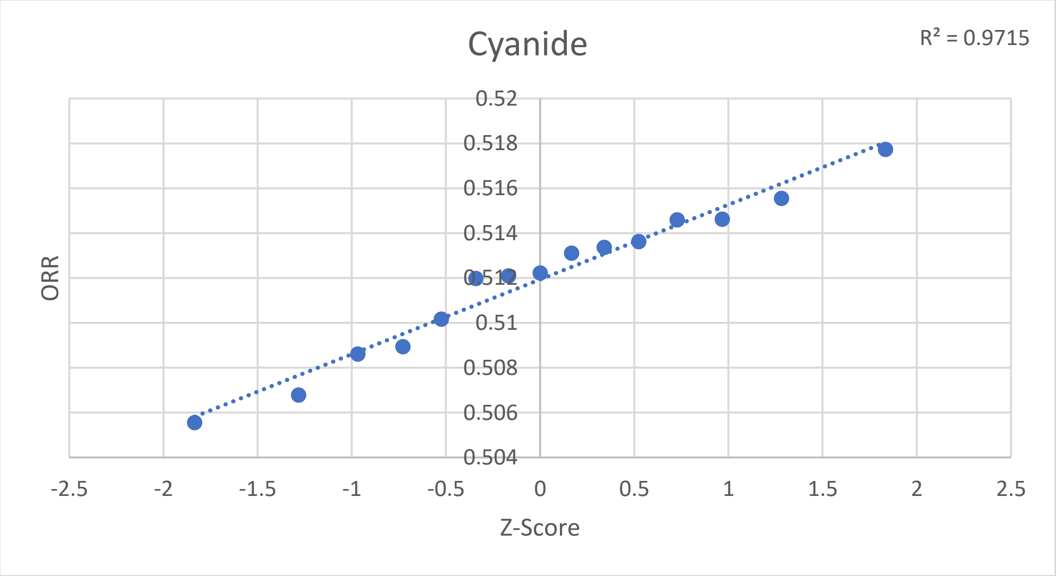

4.14 mW: No Cyanide

Shapiro Wilk Results: p=0.4574, Normally Distributed

Rank	Percentile	Z Score	No cyanide
1	0.033333333	-1.833914636	0.493352699
2	0.1	-1.281551566	0.493598692
3	0.166666667	-0.967421566	0.494136278
4	0.233333333	-0.727913291	0.494514567
5	0.3	-0.524400513	0.49516834
6	0.366666667	-0.340694827	0.495287527
7	0.433333333	-0.167894005	0.495707701
8	0.5	0	0.496878101
9	0.566666667	0.167894005	0.496961126
10	0.633333333	0.340694827	0.497728011
11	0.7	0.524400513	0.498116168
12	0.766666667	0.727913291	0.499349679
13	0.833333333	0.967421566	0.499772394
14	0.9	1.281551566	0.500196257
15	0.966666667	1.833914636	0.501077091

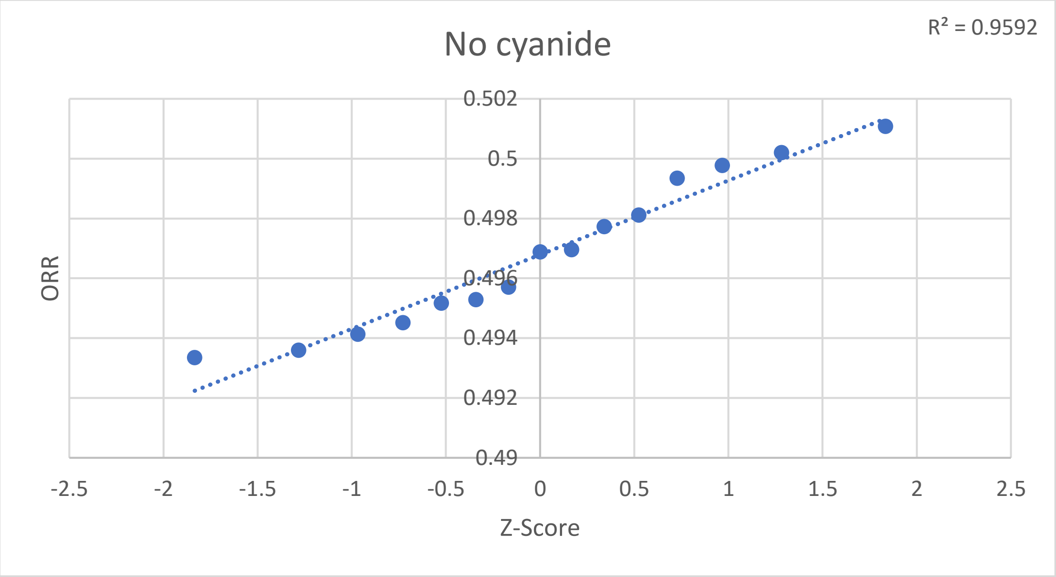

4.14 mW: Cyanide

Shapiro Wilk Results: p=0.2207, Normally Distributed

Rank	Percentile	Z Score	Cyanide
1	0.033333333	-1.833914636	0.508848904
2	0.1	-1.281551566	0.513970536
3	0.166666667	-0.967421566	0.515784053
4	0.233333333	-0.727913291	0.516314652
5	0.3	-0.524400513	0.520040095
6	0.366666667	-0.340694827	0.523755886
7	0.433333333	-0.167894005	0.52425841
8	0.5	0	0.524652113
9	0.566666667	0.167894005	0.525537668
10	0.633333333	0.340694827	0.525736028
11	0.7	0.524400513	0.525768693
12	0.766666667	0.727913291	0.527033522
13	0.833333333	0.967421566	0.528006849
14	0.9	1.281551566	0.528412564
15	0.966666667	1.833914636	0.538105396

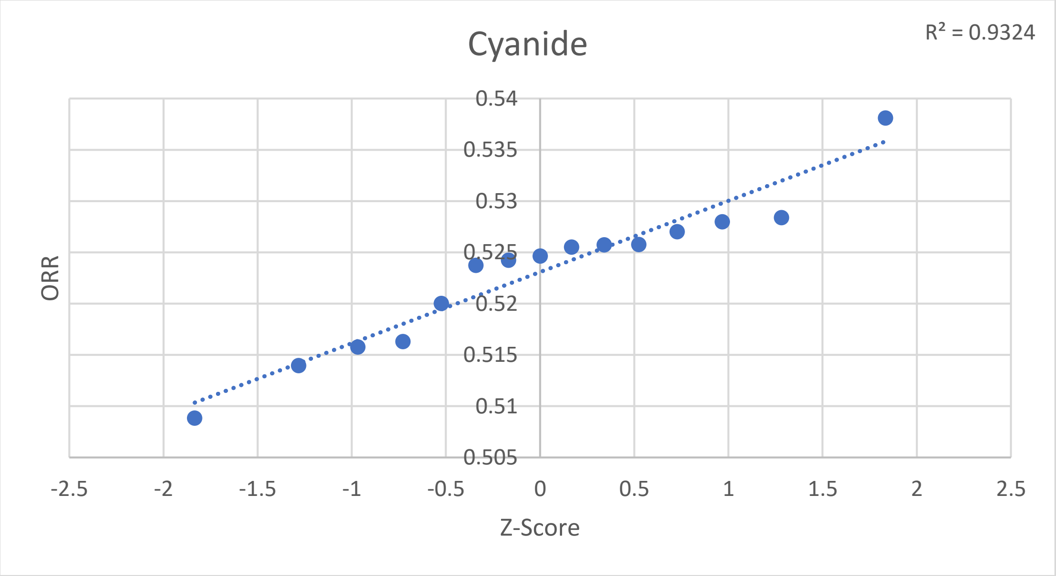

7.90 mW: No Cyanide			
Shapiro Wilk Results: p= 0.3687, Normally Distributed			
Rank	Percentile	Z Score	No cyanide
1	0.033333333	-1.833914636	0.492829812
2	0.1	-1.281551566	0.495765763
3	0.166666667	-0.967421566	0.497302795
4	0.233333333	-0.727913291	0.497739238
5	0.3	-0.524400513	0.498297797
6	0.366666667	-0.340694827	0.49859597
7	0.433333333	-0.167894005	0.499803709
8	0.5	0	0.500489789
9	0.566666667	0.167894005	0.504497286
10	0.633333333	0.340694827	0.504593541
11	0.7	0.524400513	0.505842608
12	0.766666667	0.727913291	0.506637697
13	0.833333333	0.967421566	0.507848361
14	0.9	1.281551566	0.508338489
15	0.966666667	1.833914636	0.509291541

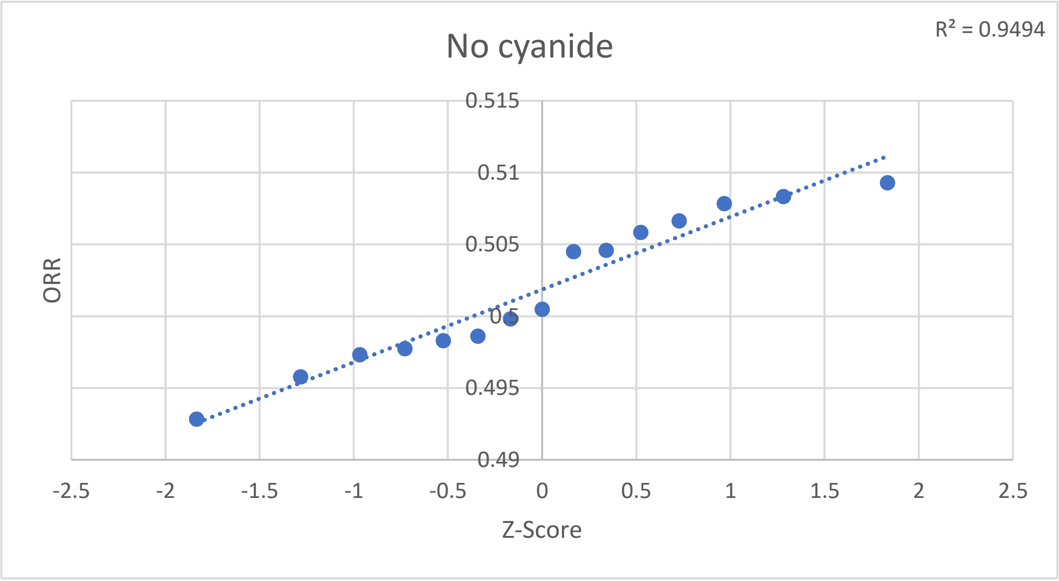

7.90 mW: Cyanide			
Shapiro Wilk Results: p= 0.8686, Normally Distributed			
Rank	Percentile	Z Score	Cyanide
1	0.033333333	-1.833914636	0.51254581
2	0.1	-1.281551566	0.517230979
3	0.166666667	-0.967421566	0.517647601
4	0.233333333	-0.727913291	0.518874404
5	0.3	-0.524400513	0.521698371
6	0.366666667	-0.340694827	0.522277638
7	0.433333333	-0.167894005	0.523108126
8	0.5	0	0.524359416
9	0.566666667	0.167894005	0.525595651
10	0.633333333	0.340694827	0.527094204
11	0.7	0.524400513	0.529463835
12	0.766666667	0.727913291	0.529905682
13	0.833333333	0.967421566	0.534083549
14	0.9	1.281551566	0.535003259
15	0.966666667	1.833914636	0.536151261

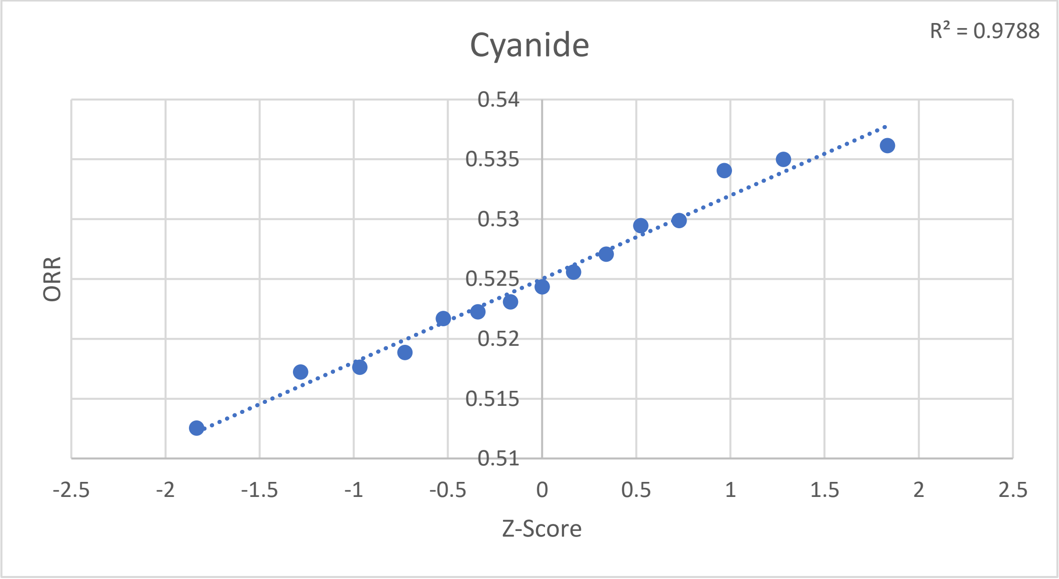

11.56 mW: No Cyanide

Shapiro Wilk Results: p= 0.8045, Normally Distributed

Rank	Percentile	Z Score	No cyanide
1	0.033333333	-1.833914636	0.493966211
2	0.1	-1.281551566	0.496907695
3	0.166666667	-0.967421566	0.496938661
4	0.233333333	-0.727913291	0.499653255
5	0.3	-0.524400513	0.500172414
6	0.366666667	-0.340694827	0.500758168
7	0.433333333	-0.167894005	0.501938118
8	0.5	0	0.503203292
9	0.566666667	0.167894005	0.504228291
10	0.633333333	0.340694827	0.504869304
11	0.7	0.524400513	0.506275143
12	0.766666667	0.727913291	0.508066962
13	0.833333333	0.967421566	0.511899121
14	0.9	1.281551566	0.513235998
15	0.966666667	1.833914636	0.516989922

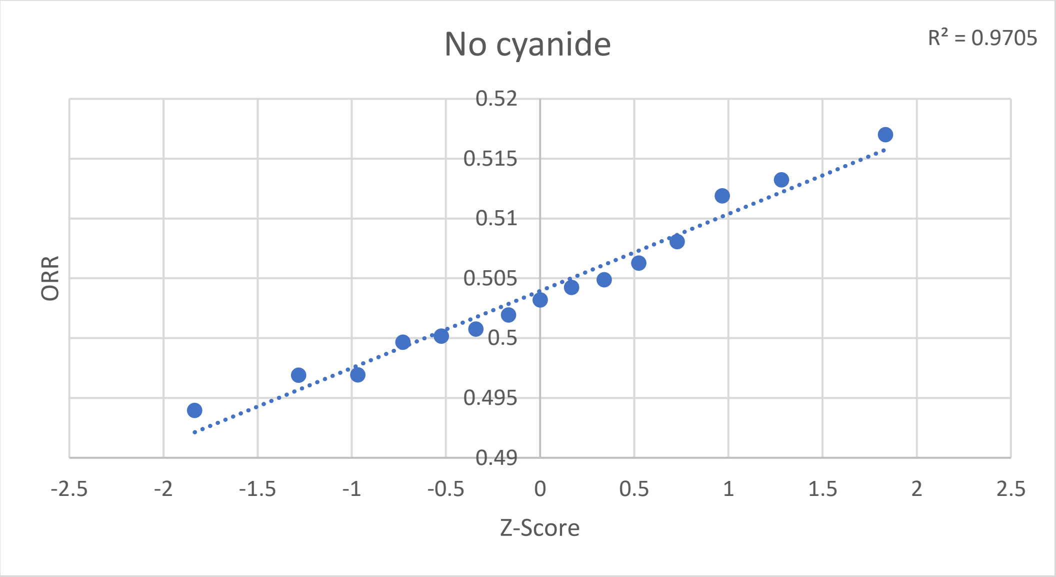

11.56 mW: Cyanide

Shapiro Wilk Results: p= 0.9440, Normally Distributed

Rank	Percentile	Z Score	Cyanide
1	0.033333333	-1.833914636	0.516716636
2	0.1	-1.281551566	0.520060673
3	0.166666667	-0.967421566	0.520622969
4	0.233333333	-0.727913291	0.522169745
5	0.3	-0.524400513	0.52246268
6	0.366666667	-0.340694827	0.524782294
7	0.433333333	-0.167894005	0.525037171
8	0.5	0	0.527847028
9	0.566666667	0.167894005	0.527969185
10	0.633333333	0.340694827	0.528181967
11	0.7	0.524400513	0.529648441
12	0.766666667	0.727913291	0.530109783
13	0.833333333	0.967421566	0.531491552
14	0.9	1.281551566	0.535324289
15	0.966666667	1.833914636	0.536408028

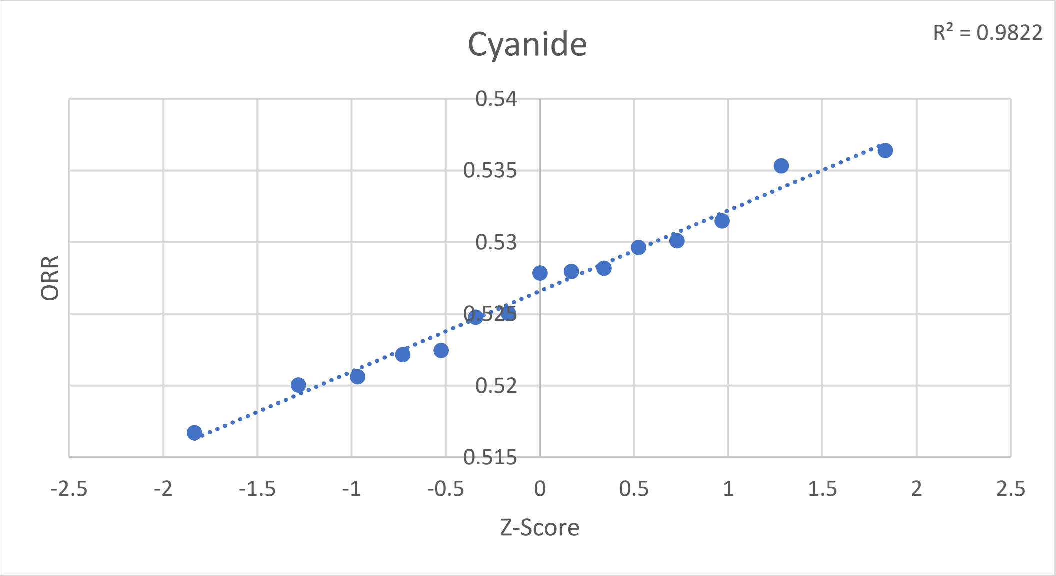

15.01 mW: No Cyanide			
Shapiro Wilk Results: p= 0.5918, Normally Distributed			
Rank	Percentile	Z Score	No cyanide
1	0.033333333	-1.833914636	0.48603178
2	0.1	-1.281551566	0.488881679
3	0.166666667	-0.967421566	0.491115835
4	0.233333333	-0.727913291	0.491781458
5	0.3	-0.524400513	0.493712786
6	0.366666667	-0.340694827	0.494751424
7	0.433333333	-0.167894005	0.495078783
8	0.5	0	0.497599646
9	0.566666667	0.167894005	0.499292983
10	0.633333333	0.340694827	0.499727842
11	0.7	0.524400513	0.501623463
12	0.766666667	0.727913291	0.501935993
13	0.833333333	0.967421566	0.503147763
14	0.9	1.281551566	0.504303858
15	0.966666667	1.833914636	0.504782238

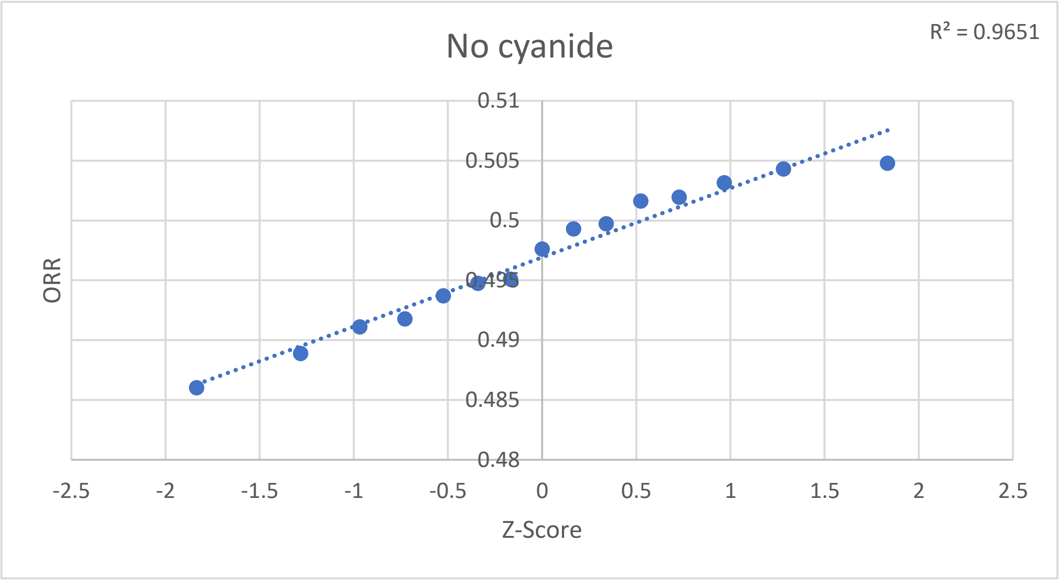

15.01 mW: Cyanide			
Shapiro Wilk Results: p= 0.4858 , Normally Distributed			
Rank	Percentile	Z Score	Cyanide
1	0.033333333	-1.833914636	0.518655452
2	0.1	-1.281551566	0.519338467
3	0.166666667	-0.967421566	0.524793515
4	0.233333333	-0.727913291	0.526889239
5	0.3	-0.524400513	0.530797273
6	0.366666667	-0.340694827	0.531536368
7	0.433333333	-0.167894005	0.533082316
8	0.5	0	0.539592172
9	0.566666667	0.167894005	0.540481908
10	0.633333333	0.340694827	0.541735125
11	0.7	0.524400513	0.542437829
12	0.766666667	0.727913291	0.54262838
13	0.833333333	0.967421566	0.544432274
14	0.9	1.281551566	0.548567126
15	0.966666667	1.833914636	0.551424689

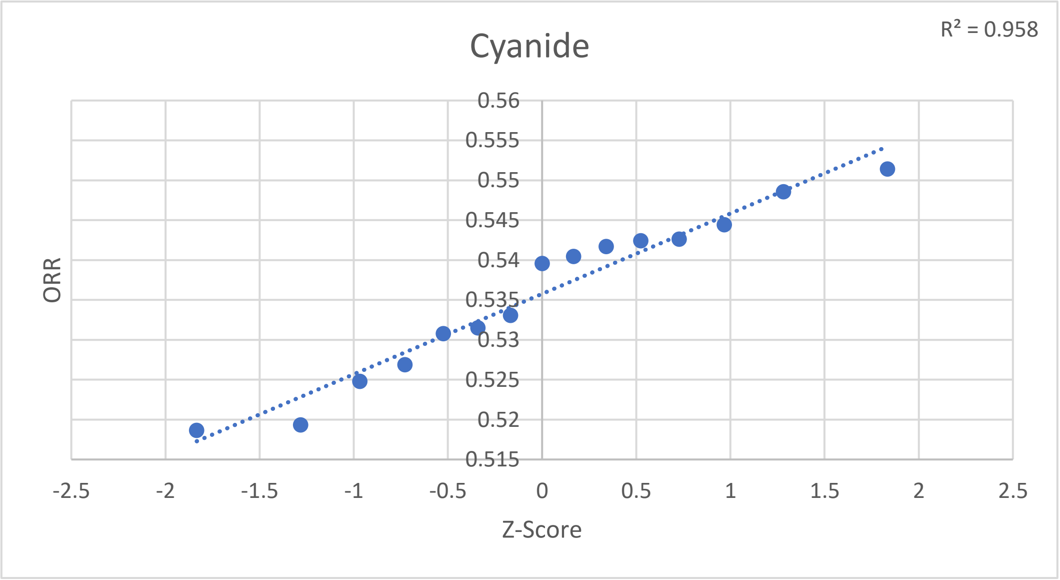

18.29 mW: No Cyanide

Shapiro Wilk Results: p=0.5743, Normally Distributed

Rank	Percentile	Z Score	No cyanide
1	0.033333333	-1.833914636	0.472199601
2	0.1	-1.281551566	0.480613721
3	0.166666667	-0.967421566	0.483537133
4	0.233333333	-0.727913291	0.484209968
5	0.3	-0.524400513	0.484441285
6	0.366666667	-0.340694827	0.48460057
7	0.433333333	-0.167894005	0.484658426
8	0.5	0	0.486650891
9	0.566666667	0.167894005	0.487681825
10	0.633333333	0.340694827	0.491776629
11	0.7	0.524400513	0.495487532
12	0.766666667	0.727913291	0.496507149
13	0.833333333	0.967421566	0.498876515
14	0.9	1.281551566	0.503007843
15	0.966666667	1.833914636	0.509099041

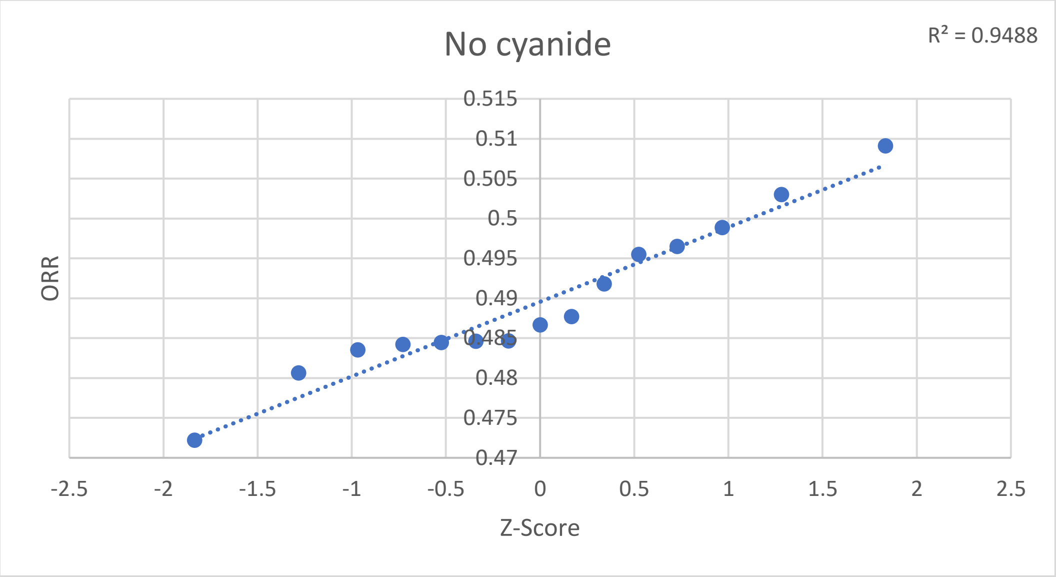

18.29 mW: Cyanide

Shapiro Wilk Results: p=0.2753, Normally Distributed

Rank	Percentile	Z Score	Cyanide
1	0.033333333	-1.833914636	0.521639423
2	0.1	-1.281551566	0.523281809
3	0.166666667	-0.967421566	0.527440186
4	0.233333333	-0.727913291	0.52764036
5	0.3	-0.524400513	0.530473952
6	0.366666667	-0.340694827	0.531408174
7	0.433333333	-0.167894005	0.535815044
8	0.5	0	0.538090797
9	0.566666667	0.167894005	0.542225759
10	0.633333333	0.340694827	0.547650705
11	0.7	0.524400513	0.550273441
12	0.766666667	0.727913291	0.551867244
13	0.833333333	0.967421566	0.552107244
14	0.9	1.281551566	0.553498828
15	0.966666667	1.833914636	0.558672681

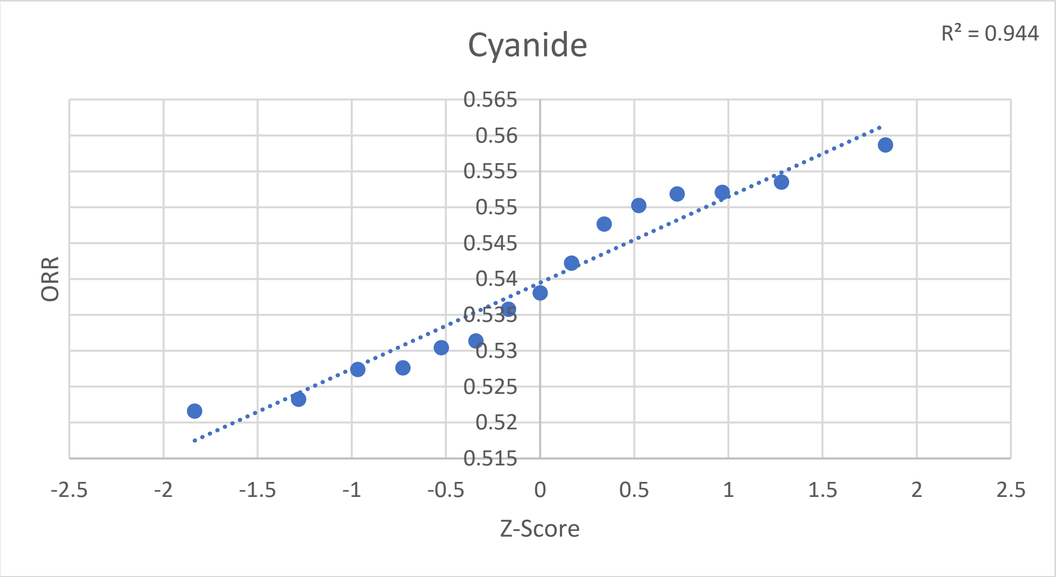

QQ-Plots for Exposure Assessment: MCF-7

5 ms: No Cyanide				
Shapiro Wilk Results: p=0.5621, Normally Distributed				
Rank	Percentile	Z Score	No cyanide	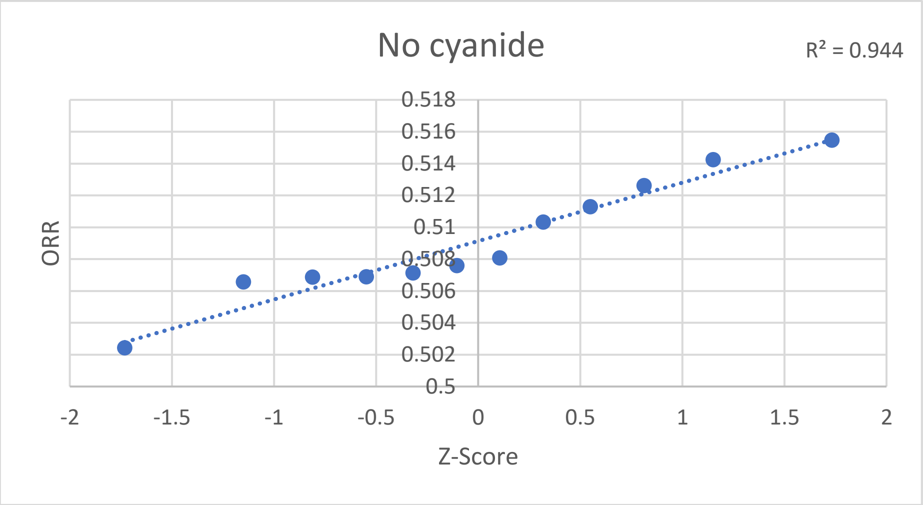
1	0.041666667	-1.731664396	0.502435128	
2	0.125	-1.15034938	0.50657556	
3	0.208333333	-0.812217801	0.506879017	
4	0.291666667	-0.548522283	0.506903234	
5	0.375	-0.318639364	0.507149445	
6	0.458333333	-0.104633456	0.507592793	
7	0.541666667	0.104633456	0.508077148	
8	0.625	0.318639364	0.510333269	
9	0.708333333	0.548522283	0.511293023	
10	0.791666667	0.812217801	0.512620144	
11	0.875	1.15034938	0.514246766	
12	0.958333333	1.731664396	0.515484319	

5 ms: Cyanide				
Shapiro Wilk Results: p=0.7140, Normally Distributed				
Rank	Percentile	Z Score	Cyanide	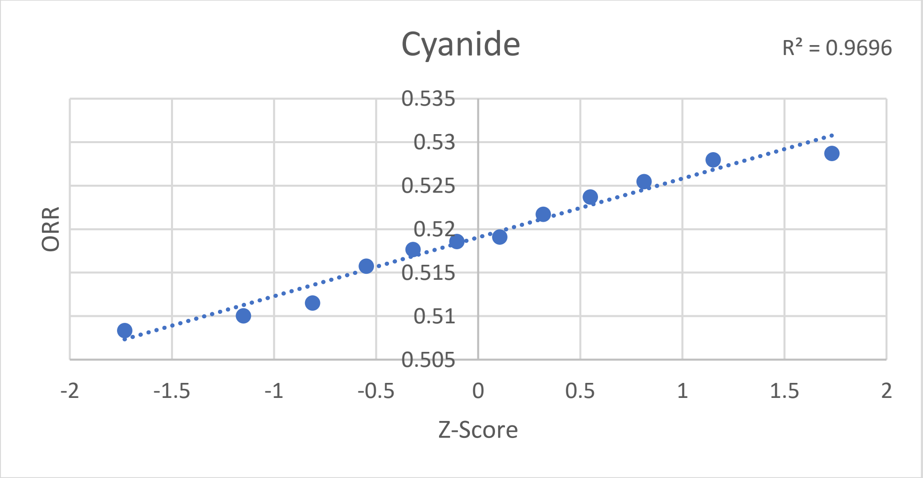
1	0.041666667	-1.731664396	0.508346585	
2	0.125	-1.15034938	0.510029437	
3	0.208333333	-0.812217801	0.511534208	
4	0.291666667	-0.548522283	0.515753214	
5	0.375	-0.318639364	0.51765477	
6	0.458333333	-0.104633456	0.518595093	
7	0.541666667	0.104633456	0.519109813	
8	0.625	0.318639364	0.521727238	
9	0.708333333	0.548522283	0.52372085	
10	0.791666667	0.812217801	0.525480415	
11	0.875	1.15034938	0.527986133	
12	0.958333333	1.731664396	0.528715631	

10 ms: No Cyanide

Shapiro Wilk Results: p=0.1383, Normally Distributed

Rank	Percentile	Z Score	No cyanide
1	0.041666667	-1.731664396	0.509353261
2	0.125	-1.15034938	0.512291091
3	0.208333333	-0.812217801	0.513001949
4	0.291666667	-0.548522283	0.513346728
5	0.375	-0.318639364	0.518232827
6	0.458333333	-0.104633456	0.518867781
7	0.541666667	0.104633456	0.518917483
8	0.625	0.318639364	0.519236433
9	0.708333333	0.548522283	0.520744776
10	0.791666667	0.812217801	0.528969577
11	0.875	1.15034938	0.533714636
12	0.958333333	1.731664396	0.538657212

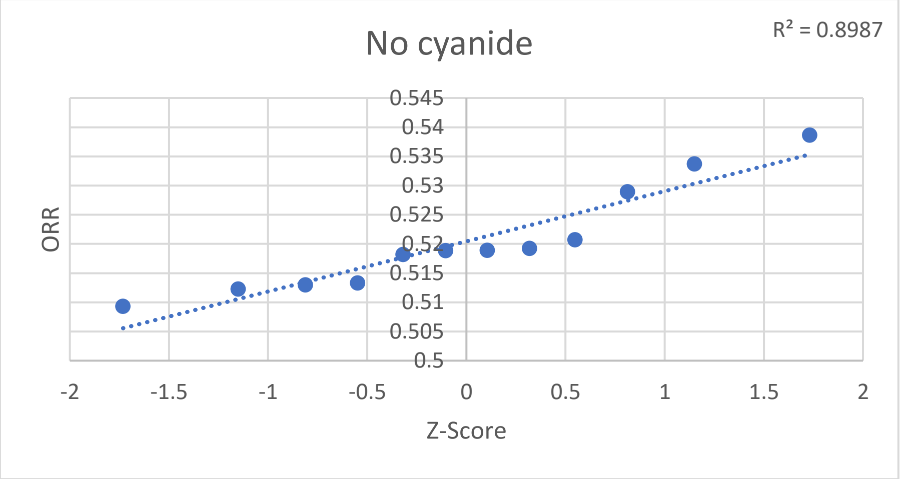

10 ms: Cyanide

Shapiro Wilk Results: p=0.3099, Normally Distributed

Rank	Percentile	Z Score	Cyanide
1	0.041666667	-1.731664396	0.527736381
2	0.125	-1.15034938	0.528085229
3	0.208333333	-0.812217801	0.531783116
4	0.291666667	-0.548522283	0.532701896
5	0.375	-0.318639364	0.533397409
6	0.458333333	-0.104633456	0.534000352
7	0.541666667	0.104633456	0.536357567
8	0.625	0.318639364	0.538978129
9	0.708333333	0.548522283	0.540109697
10	0.791666667	0.812217801	0.547003475
11	0.875	1.15034938	0.548346785
12	0.958333333	1.731664396	0.553008883

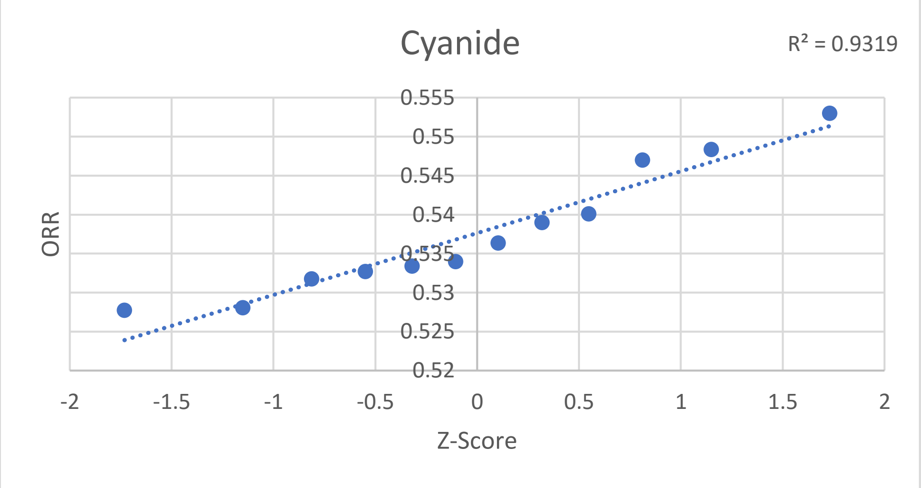

20 ms: No Cyanide			
Shapiro Wilk Results: p=0.0725, Normally Distributed			
Rank	Percentile	Z Score	No cyanide
1	0.041666667	-1.731664396	0.51580352
2	0.125	-1.15034938	0.521497249
3	0.208333333	-0.812217801	0.522166326
4	0.291666667	-0.548522283	0.523615504
5	0.375	-0.318639364	0.523976761
6	0.458333333	-0.104633456	0.529464563
7	0.541666667	0.104633456	0.531305605
8	0.625	0.318639364	0.532159382
9	0.708333333	0.548522283	0.532946846
10	0.791666667	0.812217801	0.537311456
11	0.875	1.15034938	0.551098723
12	0.958333333	1.731664396	0.561525714

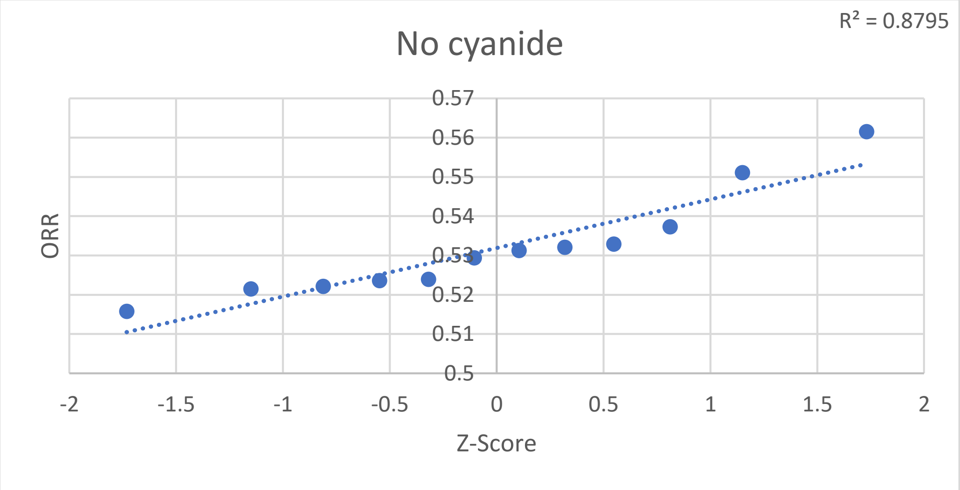

20 ms: Cyanide			
Shapiro Wilk Results: p=0.2750, Normally Distributed			
Rank	Percentile	Z Score	Cyanide
1	0.041667	-1.73166	0.545926
2	0.125	-1.15035	0.54733654
3	0.208333	-0.81222	0.55276132
4	0.291667	-0.54852	0.55480356
5	0.375	-0.31864	0.55779557
6	0.458333	-0.10463	0.55820364
7	0.541667	0.104633	0.55851576
8	0.625	0.318639	0.56168054
9	0.708333	0.548522	0.56729638
10	0.791667	0.812218	0.57515735
11	0.875	1.150349	0.57656227
12	0.958333	1.731664	0.57696462

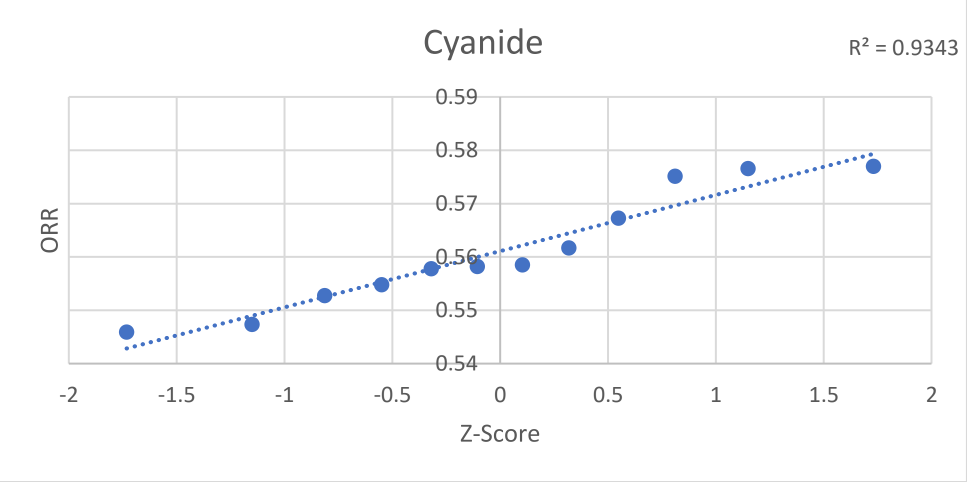

50 ms: No Cyanide

Shapiro Wilk Results: p=0.8833, Normally Distributed

Rank	Percentile	Z Score	No cyanide
1	0.041666667	-1.731664396	0.52414261
2	0.125	-1.15034938	0.538013346
3	0.208333333	-0.812217801	0.540813531
4	0.291666667	-0.548522283	0.542859809
5	0.375	-0.318639364	0.543702451
6	0.458333333	-0.104633456	0.548799958
7	0.541666667	0.104633456	0.54895847
8	0.625	0.318639364	0.552382292
9	0.708333333	0.548522283	0.553319028
10	0.791666667	0.812217801	0.557812067
11	0.875	1.15034938	0.563535342
12	0.958333333	1.731664396	0.565165725

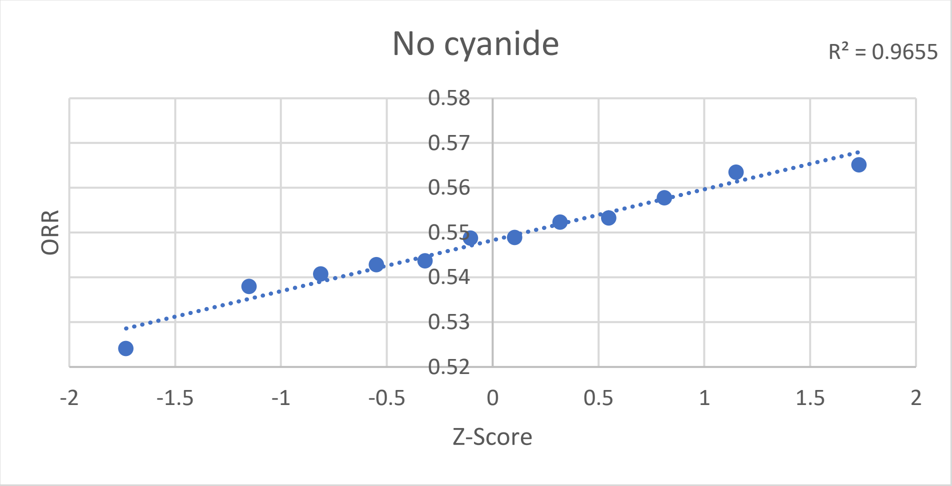

50 ms: Cyanide

Shapiro Wilk Results: p=0.2647, Normally Distributed

Rank	Percentile	Z Score	Cyanide
1	0.041666667	-1.731664396	0.576211039
2	0.125	-1.15034938	0.581006104
3	0.208333333	-0.812217801	0.581589057
4	0.291666667	-0.548522283	0.581877962
5	0.375	-0.318639364	0.58342899
6	0.458333333	-0.104633456	0.587015154
7	0.541666667	0.104633456	0.588548996
8	0.625	0.318639364	0.597315897
9	0.708333333	0.548522283	0.598737884
10	0.791666667	0.812217801	0.599488508
11	0.875	1.15034938	0.609001108
12	0.958333333	1.731664396	0.614652575

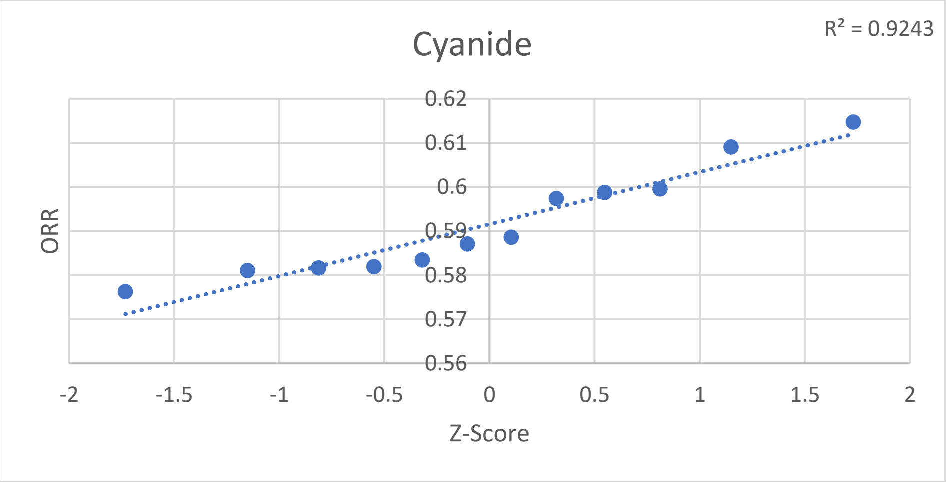

100 ms: No Cyanide			
Shapiro Wilk Results: p=0.5563, Normally Distributed			
Rank	Percentile	Z Score	No cyanide
1	0.041666667	-1.731664396	0.537367644
2	0.125	-1.15034938	0.547737439
3	0.208333333	-0.812217801	0.561394202
4	0.291666667	-0.548522283	0.562918665
5	0.375	-0.318639364	0.565431104
6	0.458333333	-0.104633456	0.567951285
7	0.541666667	0.104633456	0.568415356
8	0.625	0.318639364	0.572597218
9	0.708333333	0.548522283	0.574978947
10	0.791666667	0.812217801	0.579545605
11	0.875	1.15034938	0.582649079
12	0.958333333	1.731664396	0.586541178

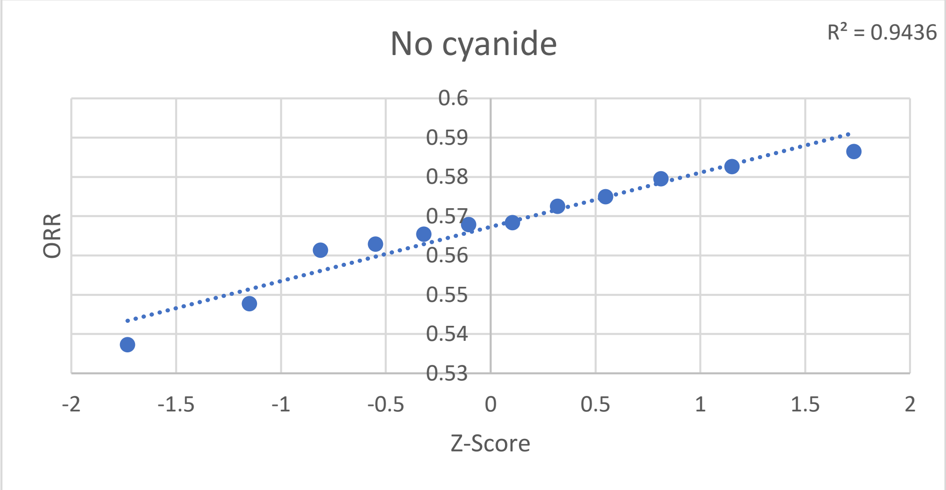

100 ms: Cyanide			
Shapiro Wilk Results: p=0.0911, Normally Distributed			
Rank	Percentile	Z Score	Cyanide
1	0.041666667	-1.731664396	0.587397335
2	0.125	-1.15034938	0.58955766
3	0.208333333	-0.812217801	0.595290079
4	0.291666667	-0.548522283	0.595461828
5	0.375	-0.318639364	0.598547351
6	0.458333333	-0.104633456	0.604852681
7	0.541666667	0.104633456	0.606016841
8	0.625	0.318639364	0.608940074
9	0.708333333	0.548522283	0.611137659
10	0.791666667	0.812217801	0.611609887
11	0.875	1.15034938	0.611615798
12	0.958333333	1.731664396	0.61233958

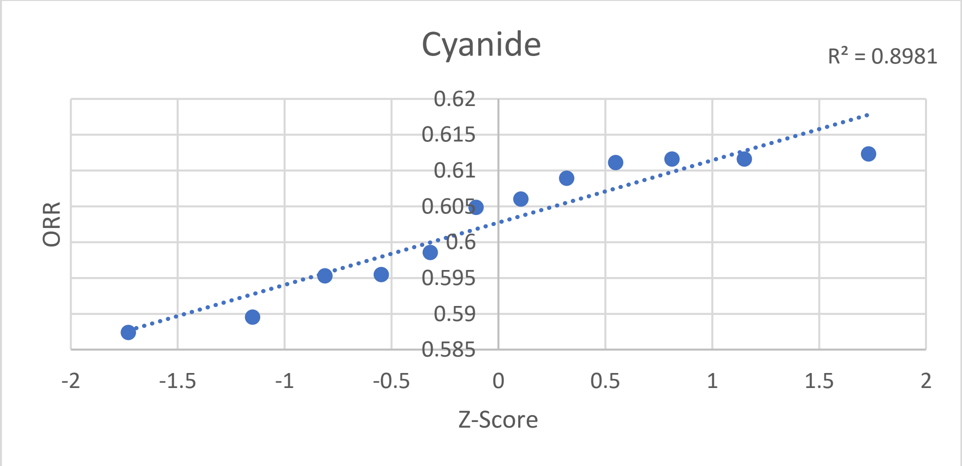

200 ms: No Cyanide			
Shapiro Wilk Results: p=0.6148, Normally Distributed			
Rank	Percentile	Z Score	No cyanide
1	0.041666667	-1.731664396	0.562666835
2	0.125	-1.15034938	0.566690922
3	0.208333333	-0.812217801	0.569853373
4	0.291666667	-0.548522283	0.57197232
5	0.375	-0.318639364	0.57867892
6	0.458333333	-0.104633456	0.582256928
7	0.541666667	0.104633456	0.583201876
8	0.625	0.318639364	0.594794925
9	0.708333333	0.548522283	0.597395051
10	0.791666667	0.812217801	0.599606852
11	0.875	1.15034938	0.6067811
12	0.958333333	1.731664396	0.625391

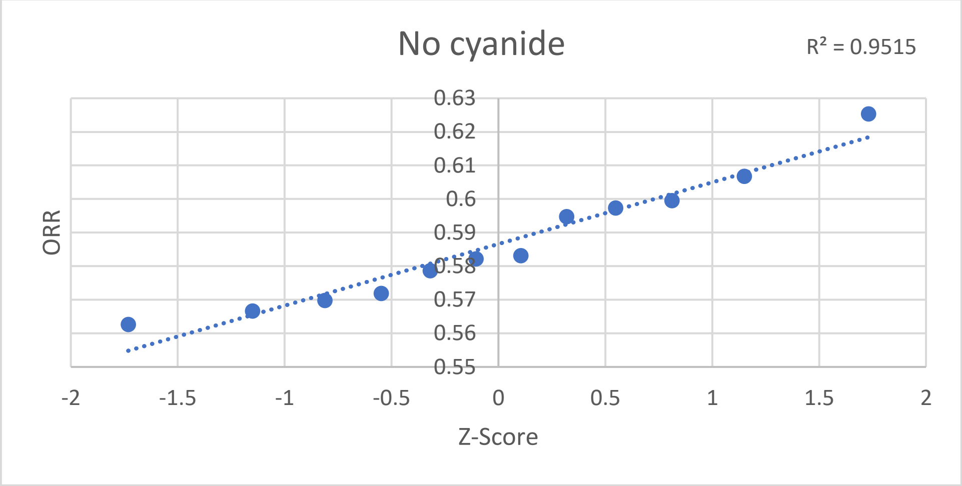

200 ms: Cyanide			
Shapiro Wilk Results: p=0.7485, Normally Distributed			
Rank	Percentile	Z Score	Cyanide
1	0.041666667	-1.731664396	0.610141372
2	0.125	-1.15034938	0.619978276
3	0.208333333	-0.812217801	0.622570053
4	0.291666667	-0.548522283	0.624155146
5	0.375	-0.318639364	0.625639425
6	0.458333333	-0.104633456	0.630381834
7	0.541666667	0.104633456	0.630752713
8	0.625	0.318639364	0.636160431
9	0.708333333	0.548522283	0.638772219
10	0.791666667	0.812217801	0.644429216
11	0.875	1.15034938	0.64726435
12	0.958333333	1.731664396	0.647530954

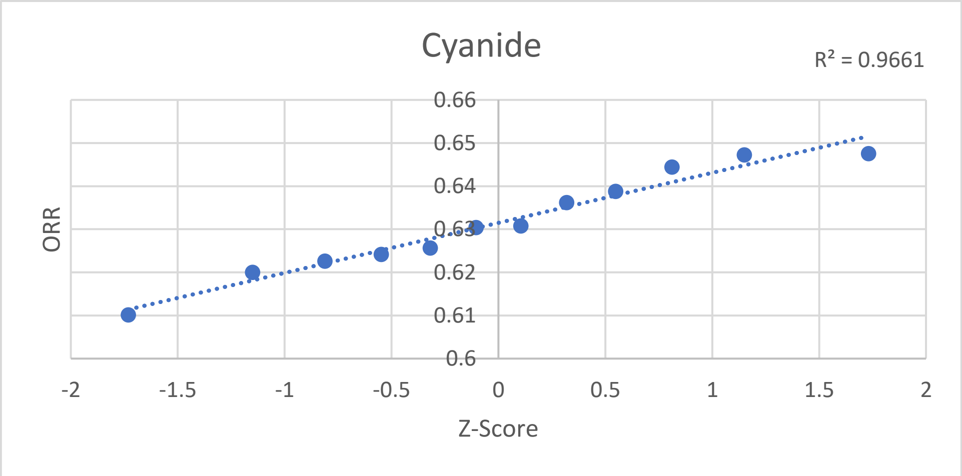

500 ms: No Cyanide

Shapiro Wilk Results: p=0.4574, Normally Distributed

Rank	Percentile	Z Score	No cyanide
1	0.041667	-1.73166	0.552227
2	0.125	-1.15035	0.574475
3	0.208333	-0.81222	0.57622
4	0.291667	-0.54852	0.581118
5	0.375	-0.31864	0.582383
6	0.458333	-0.10463	0.582972
7	0.541667	0.104633	0.585654
8	0.625	0.318639	0.587603
9	0.708333	0.548522	0.594588
10	0.791667	0.812218	0.599993
11	0.875	1.150349	0.602151
12	0.958333	1.731664	0.618159

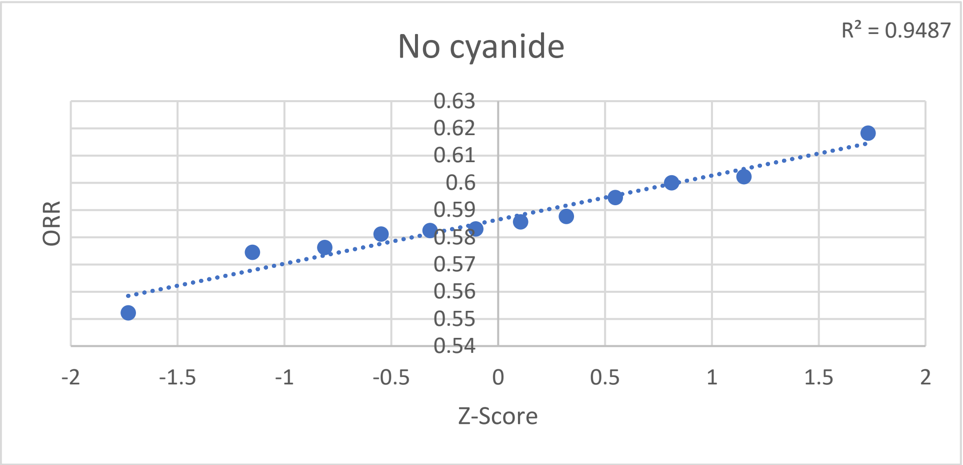

500 ms: Cyanide

Shapiro Wilk Results: p=0.3872, Normally Distributed

Rank	Percentile	Z Score	Cyanide
1	0.041667	-1.73166	0.61970561
2	0.125	-1.15035	0.62401401
3	0.208333	-0.81222	0.62409828
4	0.291667	-0.54852	0.62470546
5	0.375	-0.31864	0.62479646
6	0.458333	-0.10463	0.62966475
7	0.541667	0.104633	0.63267987
8	0.625	0.318639	0.63763245
9	0.708333	0.548522	0.6377126
10	0.791667	0.812218	0.64185482
11	0.875	1.150349	0.64200953
12	0.958333	1.731664	0.65034436

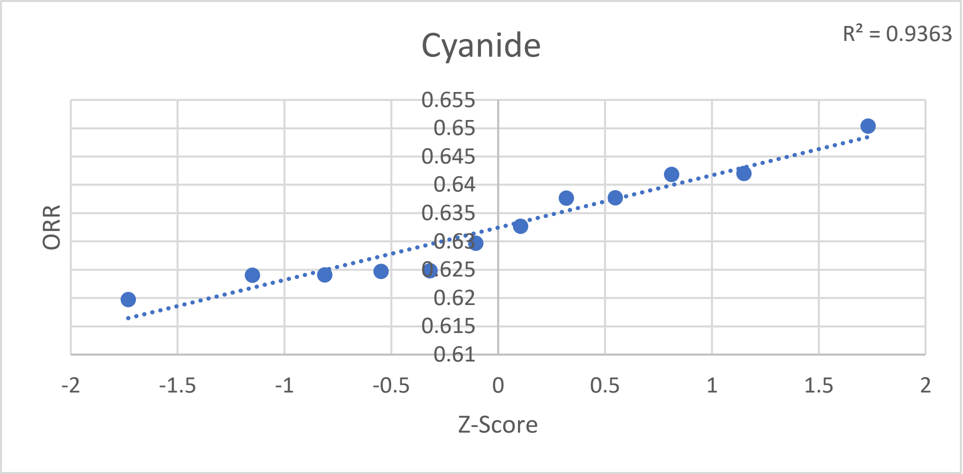

QQ-Plots for Exposure Assessment: Neurons

5 ms: No Cyanide			
Shapiro Wilk Results: p=0.0745, Normally Distributed			
Rank	Percentile	Z Score	No cyanide
1	0.041666667	-1.731664396	0.499785053
2	0.125	-1.15034938	0.50015405
3	0.208333333	-0.812217801	0.502011771
4	0.291666667	-0.548522283	0.502028111
5	0.375	-0.318639364	0.502255321
6	0.458333333	-0.104633456	0.502280649
7	0.541666667	0.104633456	0.502315644
8	0.625	0.318639364	0.502561123
9	0.708333333	0.548522283	0.50340555
10	0.791666667	0.812217801	0.506012623
11	0.875	1.15034938	0.50640105
12	0.958333333	1.731664396	0.506537914

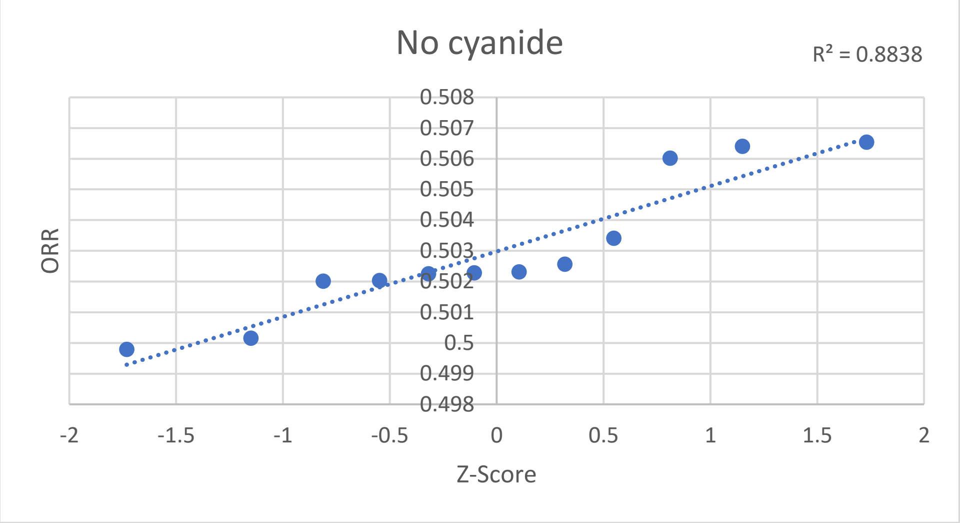

5 ms: Cyanide			
Shapiro Wilk Results: p=0.4833, Normally Distributed			
Rank	Percentile	Z Score	Cyanide
1	0.041666667	-1.731664396	0.502307628
2	0.125	-1.15034938	0.502820551
3	0.208333333	-0.812217801	0.504611038
4	0.291666667	-0.548522283	0.50496513
5	0.375	-0.318639364	0.505075363
6	0.458333333	-0.104633456	0.505221263
7	0.541666667	0.104633456	0.505268038
8	0.625	0.318639364	0.505412408
9	0.708333333	0.548522283	0.506836334
10	0.791666667	0.812217801	0.507268607
11	0.875	1.15034938	0.508427451
12	0.958333333	1.731664396	0.508625595

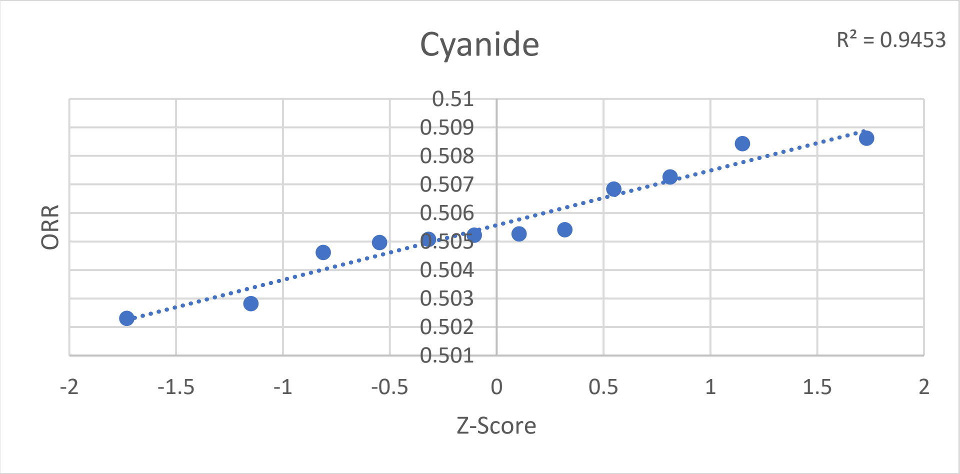

10 ms: No Cyanide

Shapiro Wilk Results: p=0.0138, Not normally Distributed

Rank	Percentile	Z Score	No cyanide
1	0.041666667	-1.731664396	0.499101006
2	0.125	-1.15034938	0.501311022
3	0.208333333	-0.812217801	0.502172115
4	0.291666667	-0.548522283	0.502601123
5	0.375	-0.318639364	0.502627254
6	0.458333333	-0.104633456	0.502735635
7	0.541666667	0.104633456	0.50354387
8	0.625	0.318639364	0.50547558
9	0.708333333	0.548522283	0.50705622
10	0.791666667	0.812217801	0.507598772
11	0.875	1.15034938	0.510246615
12	0.958333333	1.731664396	0.519682646

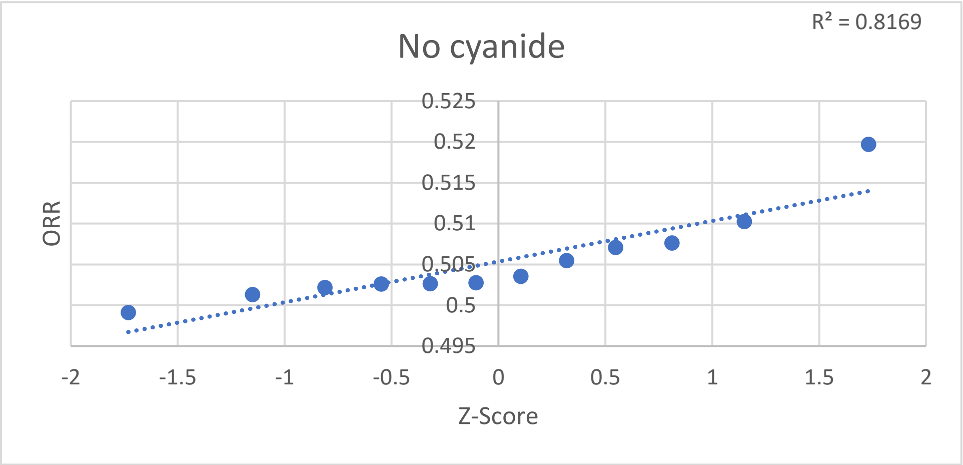

10 ms: Cyanide

Shapiro Wilk Results: p=0.1894, Normally Distributed

Rank	Percentile	Z Score	Cyanide
1	0.041666667	-1.731664396	0.505227024
2	0.125	-1.15034938	0.507004507
3	0.208333333	-0.812217801	0.507114456
4	0.291666667	-0.548522283	0.507835783
5	0.375	-0.318639364	0.507885966
6	0.458333333	-0.104633456	0.508085072
7	0.541666667	0.104633456	0.509599064
8	0.625	0.318639364	0.510013632
9	0.708333333	0.548522283	0.510193424
10	0.791666667	0.812217801	0.510883161
11	0.875	1.15034938	0.512185822
12	0.958333333	1.731664396	0.516190147

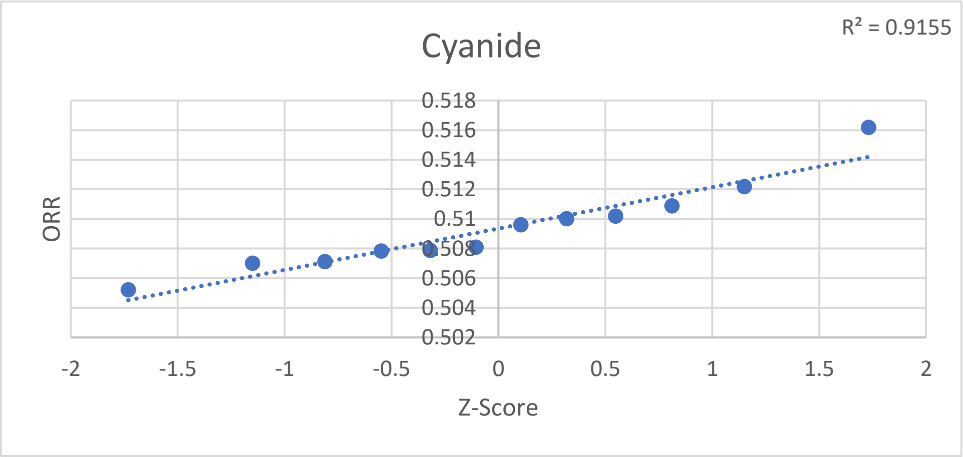

20 ms: No Cyanide

Shapiro Wilk Results: p=0.7441, Normally Distributed

Rank	Percentile	Z Score	No cyanide
1	0.041666667	-1.731664396	0.496390496
2	0.125	-1.15034938	0.498303442
3	0.208333333	-0.812217801	0.502567013
4	0.291666667	-0.548522283	0.503757758
5	0.375	-0.318639364	0.505276164
6	0.458333333	-0.104633456	0.506312268
7	0.541666667	0.104633456	0.50656226
8	0.625	0.318639364	0.507317674
9	0.708333333	0.548522283	0.508897566
10	0.791666667	0.812217801	0.512347514
11	0.875	1.15034938	0.51661376
12	0.958333333	1.731664396	0.516827229

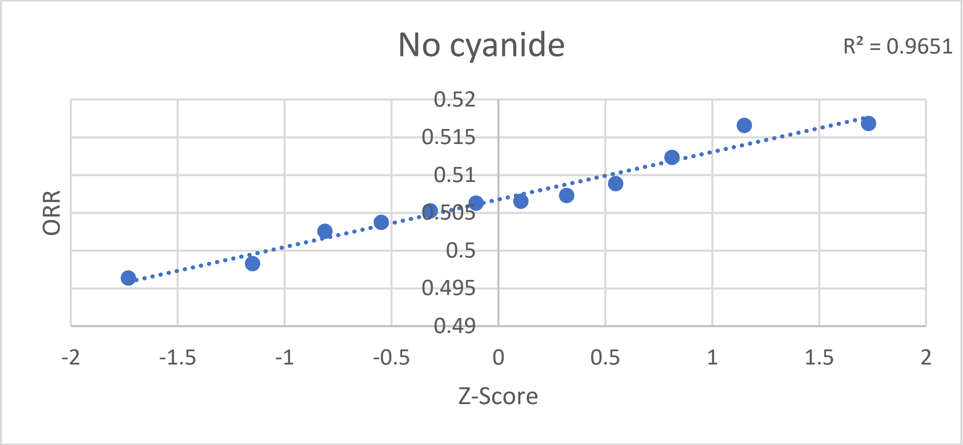

20 ms: Cyanide

Shapiro Wilk Results: p=0.4432, Normally Distributed

Rank	Percentile	Z Score	Cyanide
1	0.041667	-1.73166	0.504503987
2	0.125	-1.15035	0.510682121
3	0.208333	-0.81222	0.511064122
4	0.291667	-0.54852	0.513050391
5	0.375	-0.31864	0.513170167
6	0.458333	-0.10463	0.515085909
7	0.541667	0.104633	0.516801204
8	0.625	0.318639	0.520756935
9	0.708333	0.548522	0.520985708
10	0.791667	0.812218	0.521365615
11	0.875	1.150349	0.523997532
12	0.958333	1.731664	0.534348291

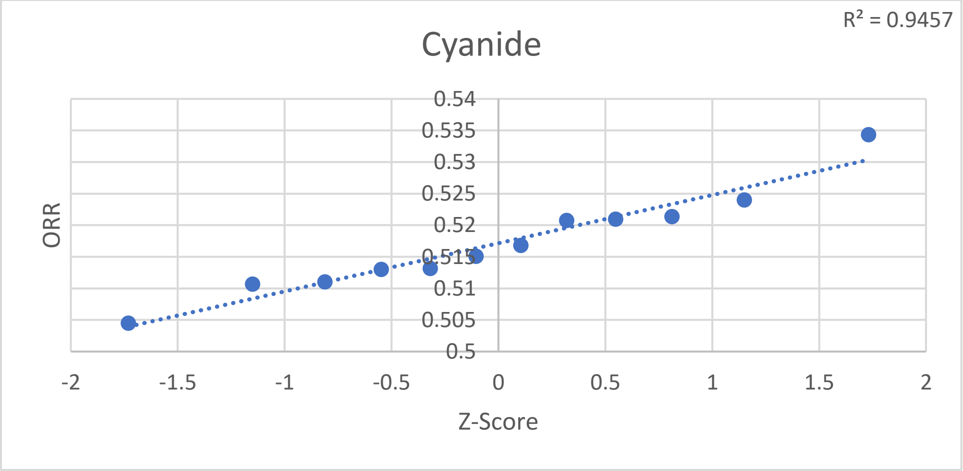

50 ms: No Cyanide

Shapiro Wilk Results: p=0.9977, Normally Distributed

Rank	Percentile	Z Score	No cyanide
1	0.041666667	-1.731664396	0.497401267
2	0.125	-1.15034938	0.505113092
3	0.208333333	-0.812217801	0.506102651
4	0.291666667	-0.548522283	0.507326972
5	0.375	-0.318639364	0.508037727
6	0.458333333	-0.104633456	0.509610194
7	0.541666667	0.104633456	0.512181294
8	0.625	0.318639364	0.513200125
9	0.708333333	0.548522283	0.514032028
10	0.791666667	0.812217801	0.516645358
11	0.875	1.15034938	0.517732448
12	0.958333333	1.731664396	0.524260965

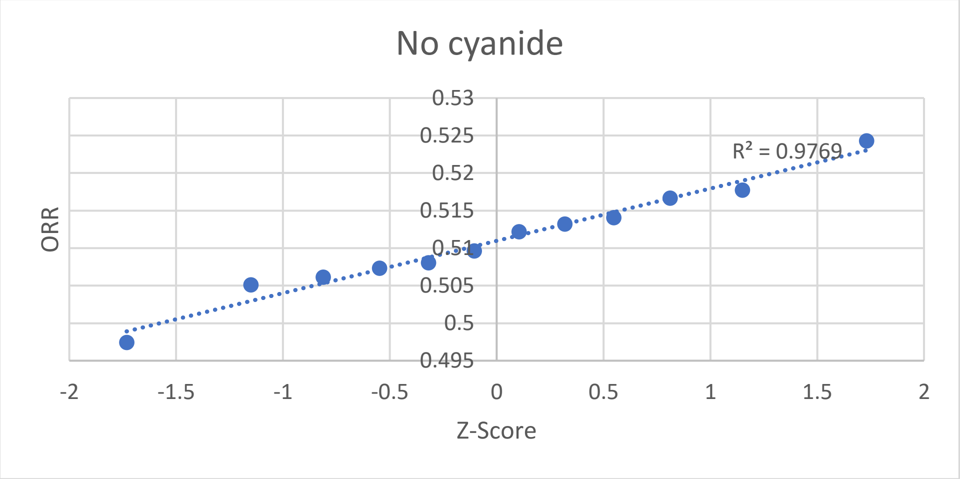

50 ms: Cyanide

Shapiro Wilk Results: p=0.4403, Normally Distributed

Rank	Percentile	Z Score	Cyanide
1	0.041666667	-1.731664396	0.515683175
2	0.125	-1.15034938	0.518453676
3	0.208333333	-0.812217801	0.528099637
4	0.291666667	-0.548522283	0.532646803
5	0.375	-0.318639364	0.535427887
6	0.458333333	-0.104633456	0.53717972
7	0.541666667	0.104633456	0.544311566
8	0.625	0.318639364	0.558872447
9	0.708333333	0.548522283	0.562120227
10	0.791666667	0.812217801	0.562844699
11	0.875	1.15034938	0.576924648
12	0.958333333	1.731664396	0.605177702

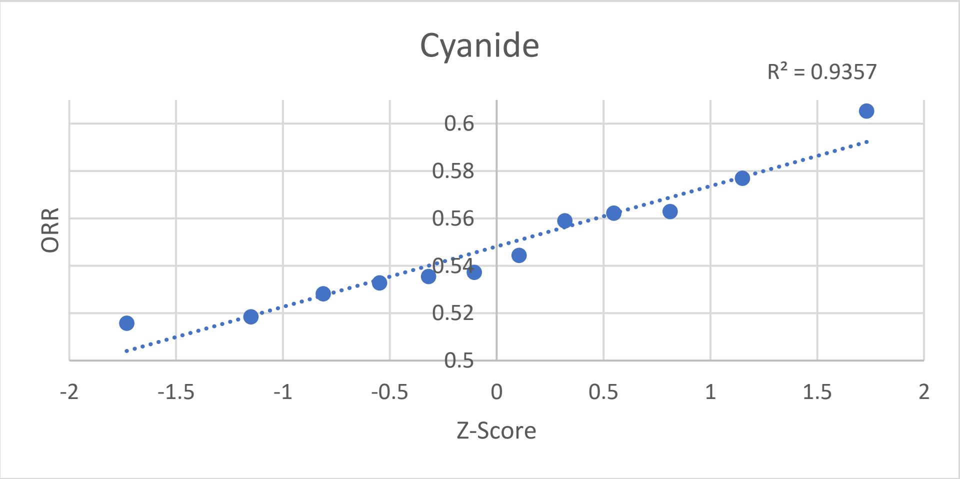

100 ms: No Cyanide

Shapiro Wilk Results: p=0.9876, Normally Distributed

Rank	Percentile	Z Score	No cyanide
1	0.041666667	-1.731664396	0.505568136
2	0.125	-1.15034938	0.510302949
3	0.208333333	-0.812217801	0.513296198
4	0.291666667	-0.548522283	0.516031284
5	0.375	-0.318639364	0.516666752
6	0.458333333	-0.104633456	0.520253611
7	0.541666667	0.104633456	0.521898181
8	0.625	0.318639364	0.522792183
9	0.708333333	0.548522283	0.527716176
10	0.791666667	0.812217801	0.53058806
11	0.875	1.15034938	0.531150547
12	0.958333333	1.731664396	0.536076374

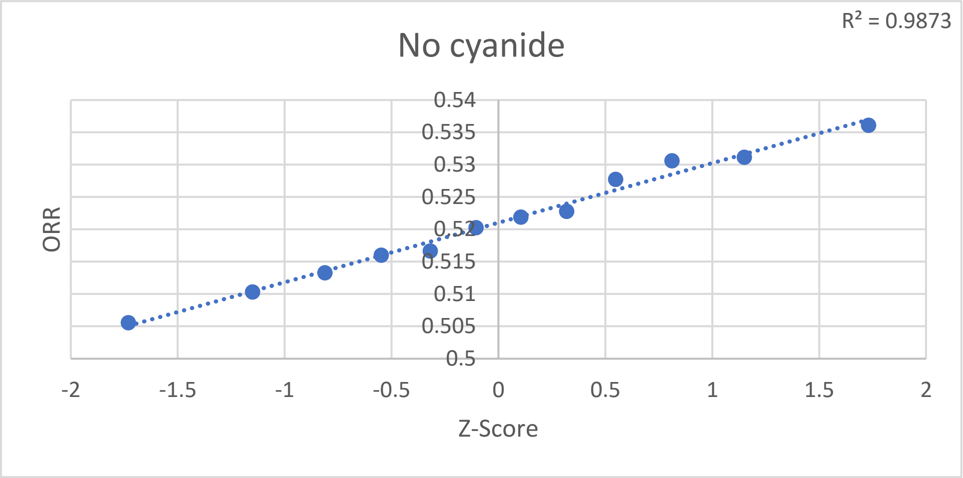

100 ms: Cyanide

Shapiro Wilk Results: p=0.9762, Normally Distributed

Rank	Percentile	Z Score	Cyanide
1	0.041666667	-1.731664396	0.544273065
2	0.125	-1.15034938	0.556485286
3	0.208333333	-0.812217801	0.562686419
4	0.291666667	-0.548522283	0.564123595
5	0.375	-0.318639364	0.565463178
6	0.458333333	-0.104633456	0.567046931
7	0.541666667	0.104633456	0.573566493
8	0.625	0.318639364	0.578055406
9	0.708333333	0.548522283	0.582568267
10	0.791666667	0.812217801	0.582850882
11	0.875	1.15034938	0.594440098
12	0.958333333	1.731664396	0.599250454

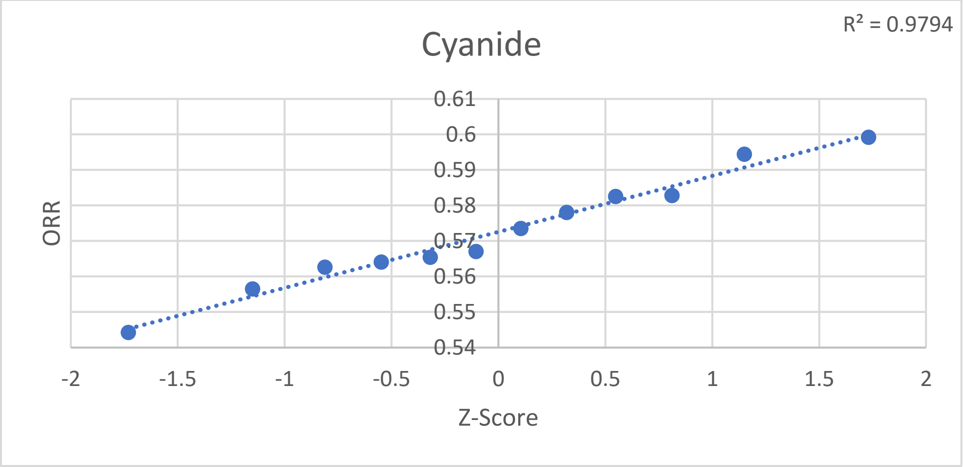

200 ms: No Cyanide			
Shapiro Wilk Results: p=0.0302, Not normally Distributed			
Rank	Percentile	Z Score	No cyanide
1	0.041666667	-1.731664396	0.519760557
2	0.125	-1.15034938	0.519809527
3	0.208333333	-0.812217801	0.520431262
4	0.291666667	-0.548522283	0.523912832
5	0.375	-0.318639364	0.527042947
6	0.458333333	-0.104633456	0.533306478
7	0.541666667	0.104633456	0.533805792
8	0.625	0.318639364	0.535391306
9	0.708333333	0.548522283	0.536034749
10	0.791666667	0.812217801	0.538471761
11	0.875	1.15034938	0.560741064
12	0.958333333	1.731664396	0.563630578

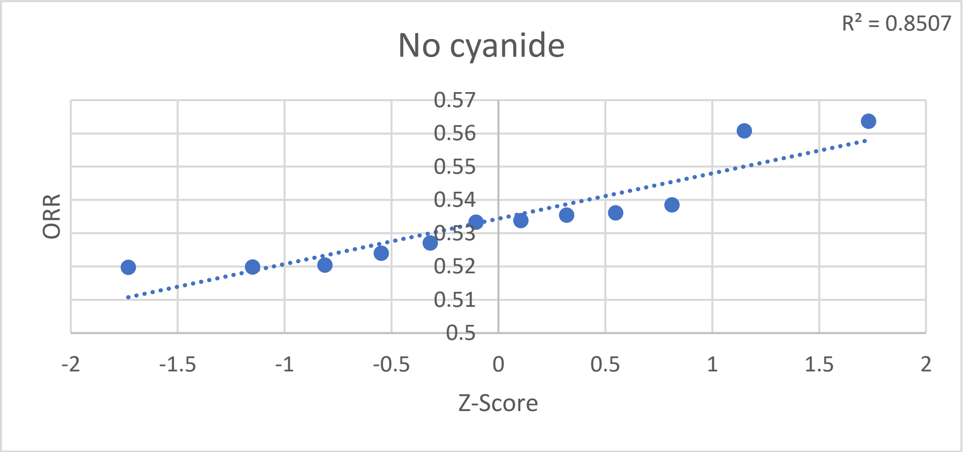

200 ms: Cyanide			
Shapiro Wilk Results: p=0.9258, Normally Distributed			
Rank	Percentile	Z Score	Cyanide
1	0.041666667	-1.731664396	0.540061082
2	0.125	-1.15034938	0.544850725
3	0.208333333	-0.812217801	0.557486594
4	0.291666667	-0.548522283	0.558174175
5	0.375	-0.318639364	0.558660968
6	0.458333333	-0.104633456	0.563254278
7	0.541666667	0.104633456	0.564562036
8	0.625	0.318639364	0.567108765
9	0.708333333	0.548522283	0.574132856
10	0.791666667	0.812217801	0.575487817
11	0.875	1.15034938	0.575918267
12	0.958333333	1.731664396	0.589087764

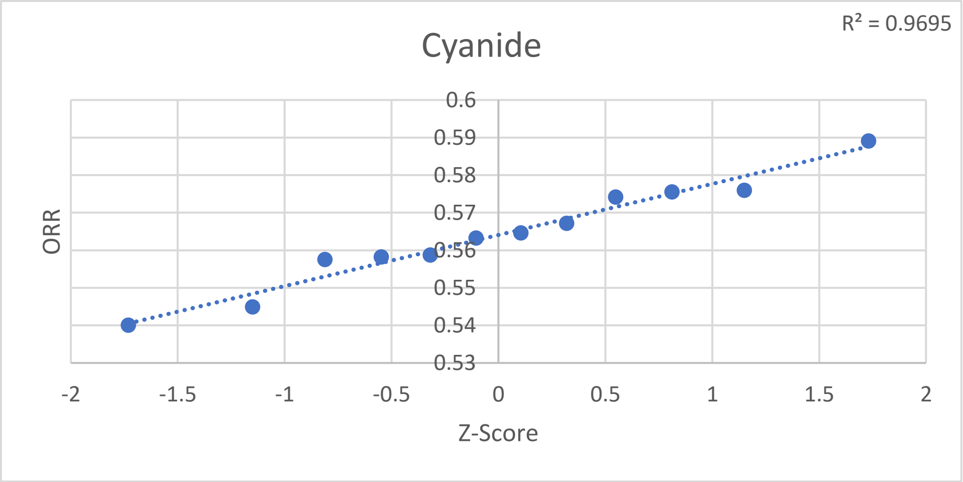

500 ms: No Cyanide			
Shapiro Wilk Results: p=0.8857, Normally Distributed			
Rank	Percentile	Z Score	No cyanide
1	0.041666667	-1.731664396	0.507417806
2	0.125	-1.15034938	0.524665584
3	0.208333333	-0.812217801	0.533325719
4	0.291666667	-0.548522283	0.535596193
5	0.375	-0.318639364	0.539333293
6	0.458333333	-0.104633456	0.542213681
7	0.541666667	0.104633456	0.543312012
8	0.625	0.318639364	0.546674805
9	0.708333333	0.548522283	0.551371663
10	0.791666667	0.812217801	0.554383819
11	0.875	1.15034938	0.563387729
12	0.958333333	1.731664396	0.57416838

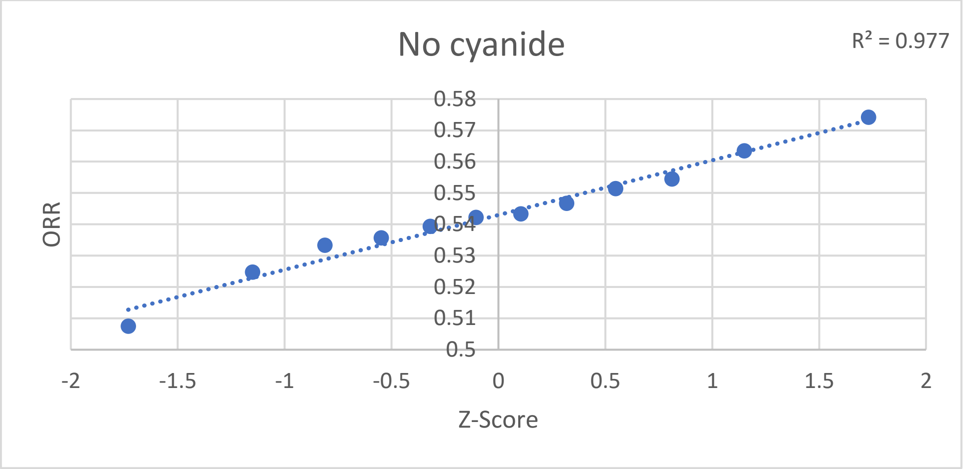

500 ms: Cyanide			
Shapiro Wilk Results: p=0.0455, Not normally Distributed			
Rank	Percentile	Z Score	Cyanide
1	0.041666667	-1.731664396	0.554121131
2	0.125	-1.15034938	0.555158295
3	0.208333333	-0.812217801	0.555861777
4	0.291666667	-0.548522283	0.556938271
5	0.375	-0.318639364	0.559033449
6	0.458333333	-0.104633456	0.562041824
7	0.541666667	0.104633456	0.562214881
8	0.625	0.318639364	0.569320389
9	0.708333333	0.548522283	0.579946961
10	0.791666667	0.812217801	0.580436874
11	0.875	1.15034938	0.586846195
12	0.958333333	1.731664396	0.58940556

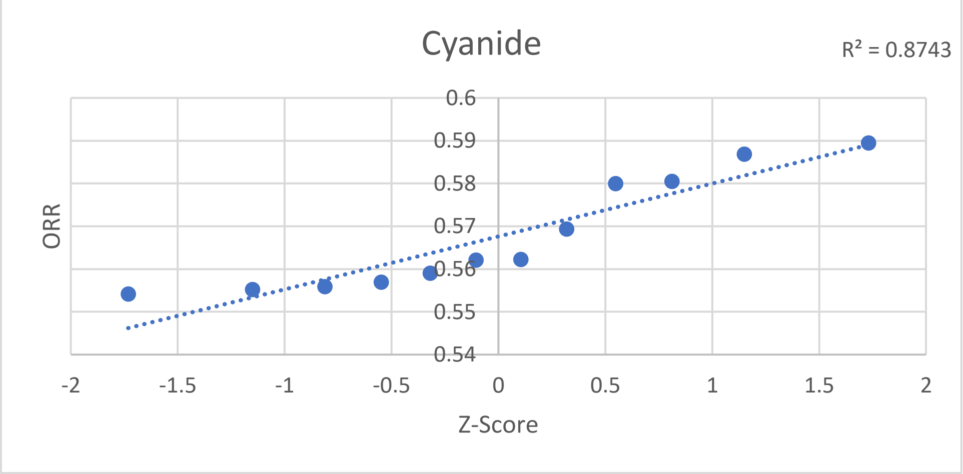

Supplement: Supplementary file 1 [file JBO_029_126501_SD001.pdf]
